# Cognitive and affective psychoeducation for Long COVID: a randomized controlled trial

**DOI:** 10.1093/braincomms/fcaf447

**Published:** 2025-11-11

**Authors:** Carmen Cabello Fernandez, Vincent Didone, Alexia Lesoinne, Hichem Slama, Patrick Fery, Anne-Françoise Rousseau, Michel Moutschen, COVCOG group, Michel Moutschen, Gilles Dupuis, Gaël Delrue, Valentine Demoulin, Julien Goin, Clara Della Libera, Fabienne Collette, Sylvie Willems

**Affiliations:** Psychology and Cognitive Neuroscience Research Unit (PsyNCog), Université de Liège (ULiège), Liège 4000, Belgium; Psychology and Cognitive Neuroscience Research Unit (PsyNCog), Université de Liège (ULiège), Liège 4000, Belgium; Psychology and Cognitive Neuroscience Research Unit (PsyNCog), Université de Liège (ULiège), Liège 4000, Belgium; Department of Neuropsychology and Speech Therapy, Université Libre de Bruxelles (ULB), Hôpital Universitaire de Bruxelles (H.U.B.), Brussels 1070, Belgium; UR2NF-Neuropsychology and Functional Neuroimaging Research Group at CRCN-Research Centre in Cognitive Neurosciences Institute, Université Libre de Bruxelles (ULB), Brussels 1050, Belgium; Department of Neuropsychology and Speech Therapy, Université Libre de Bruxelles (ULB), Hôpital Universitaire de Bruxelles (H.U.B.), Brussels 1070, Belgium; Department of Intensive Care and Burn Centre, Centre Hospitalier Universitaire de Liège, Liège 4000, Belgium; Infectious Diseases Department, Centre Hospitalier Universitaire de Liège, Liège 4000, Belgium; Psychology and Cognitive Neuroscience Research Unit (PsyNCog), Université de Liège (ULiège), Liège 4000, Belgium; GIGA-CRC Human Imaging (GIGA-CRC), Université de Liège (ULiège), Liège 4000, Belgium; Psychology and Cognitive Neuroscience Research Unit (PsyNCog), Université de Liège (ULiège), Liège 4000, Belgium

**Keywords:** post-COVID syndrome, cognitive intervention, rehabilitation, cognitive behavioural therapy, counselling

## Abstract

Long COVID is a complex condition characterized by persistent symptoms, including cognitive difficulties and fatigue, which significantly impact daily functioning. Although various intervention strategies inspired by approaches used in the rehabilitation of other neurological conditions have been developed to address these issues, evidence of their efficacy in Long COVID populations remains limited. This study aimed to compare the effectiveness for cognitive complaints of two psychoeducational interventions—one focused on cognitive difficulties and the other on affective symptoms in Long COVID patients with cognitive problems. COVCOG (Long COVID: treatment of cognitive difficulties) is a randomized controlled trial using a parallel two-group design. Long COVID patients underwent neuropsychological assessments at pre-, 2- and 8-month post-intervention. The intervention comprised four 90-min sessions of either a cognitive-focused or an affective-focused psychoeducational programme. The effects were measured on cognitive complaints (primary outcome), cognitive performance, fatigue, sleep difficulties, quality of life, psychological distress, and impact on work and daily activities (secondary outcomes). Linear mixed models (LMMs) were used. One hundred and thirty Long COVID patients were randomized. One hundred and twenty-two (mean age: 47 ± 10; 69.7% female) were included (63 in the cognitive group and 59 in the affective group). The low dropout rate (12% at 2 months and 9% at 8 months post-intervention) and the patients’ substantial active engagement—92% attended all intervention sessions—assured the feasibility of both interventions. LMM analysis revealed a statistically significant improvement with time in subjective cognitive complaints, objective cognitive performance (attention, working memory and long-term memory), quality of life, fatigue, sleep, some psychological distress subscales and work impairment (all *P*s < 0.03, with small to moderate effect sizes), but no group-by-time interaction, suggesting that trajectories did not differ between arms. However, some improvements are specific to one intervention or the other. Designed specifically for this population, both psychoeducative interventions provide insights into improving the management of Long COVID patients with cognitive problems. Longer treatment may be needed for more meaningful improvements. **Clinicaltrials.gov**: NCT05167266.

## Teaser

Cabello Fernandez *et al*. report that two psychoeducation interventions (cognitive versus affective) tailored to cognitive, functional and psychological difficulties in Long COVID were well-received and beneficial, as evidenced by a reduction in self-reported difficulties and an improvement in cognitive performance. However, the observed improvement is modest and similar in both interventions.

## Graphical Abstract

**Graphical Abstract:**
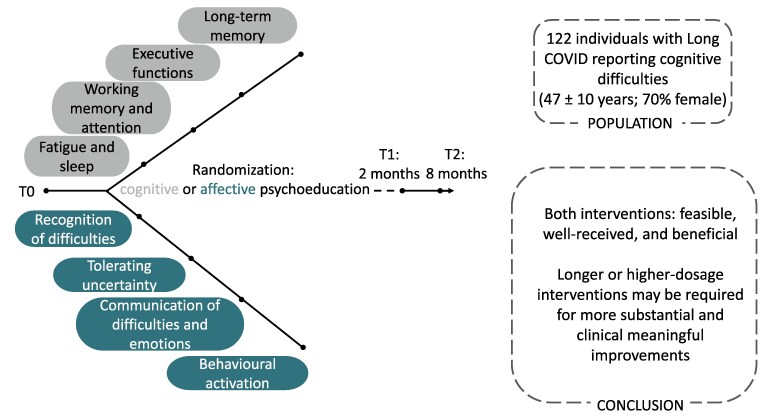

## Introduction

Long COVID is characterized by a constellation of symptoms that emerge within 3 months of a SARS-CoV-2 infection, regardless of its severity,^1^ and persist for at least 2 months.^2^ Numerous pathological mechanisms have been proposed, ranging from the consequences of acute infection to phenomena that interact systematically after the acute phase, such as viral persistence, chronic inflammation, autoimmune response, thrombotic events, mitochondrial dysfunction and metabolic perturbations (for a review, see Peluso and Deeks^3^).

Cognitive difficulties and fatigue are among the most commonly reported symptoms^1,4^; cognitive difficulties affect ∼50% of Long COVID patients.^5^ Subjective cognitive complaints (i.e. self-reported experiences of cognitive difficulties) are frequently reported in both the subacute^6^ and long-term phases,^7^ sometimes up to 24 months post-infection.^8,9^ Objective difficulties (i.e. deficits observed during standardized neuropsychological tasks) were also observed on cognitive tasks in nearly 70% of patients who reported subjective cognitive complaints.^10,11^ Objective difficulties are most often observed in attention, memory and executive functioning.^12,13^ These persistent cognitive difficulties are disabling; they negatively impact daily activities, socio-professional reintegration and psychological health and cause financial strain for the patients, their families and society.^14-16^

The first intervention programmes were inspired by established practices for other neurological conditions that provoke similar cognitive issues, such as mild to moderate traumatic brain injury (TBI).^17^ Typically, these approaches are multidimensional and centred on the patient’s goals,^18^ and address cognitive disorders directly via specific restorative training (i.e. rehabilitation focused on remediating cognitive impairment through computerized or pen-and-paper training exercises) or indirectly via metacognition (i.e. strategies to self-regulate cognitive activities), internal and external compensatory strategies (i.e. use of alternative or external aids).^19-21^ A consensus policy statement supports such combined approaches for Long COVID patients.^22^ Psychoeducation (i.e. education of the patients to implement an adequate internal representation of the disease and increase self-awareness of deficits and maladaptive behaviours) has also been proposed,^23^ as it has an important place in neuropsychological management, with interesting effects on the long-term management of difficulties in TBI.^24,25^ In this vein, Braga *et al*.,^26^ who applied a longitudinal design on 208 Long COVID patients, noted the positive effects on cognitive performance of a psychoeducational programme incorporating compensatory strategies. Similarly, using a simple pre–post design, Hasting *et al*.^27^ noted positive effects on mood and self-efficacy in managing symptoms after 10 group sessions of an intervention involving psychoeducation focused on cognitive deficits, compensatory strategies, relaxation and behavioural activation. Improved metacognition and increased use of compensatory strategies after psychoeducation focused on cognitive deficits have also been observed in case studies (e.g. Assunção *et al.*^28^).

The effectiveness of direct restorative cognitive training is still being studied (e.g. Dutra and Shigaeff^29^). Some studies report improvements in certain cognitive domains, such as memory and language.^30,31^ Nevertheless, as with other populations, the transfer of the benefits of restorative approaches into everyday life has yet to be demonstrated,^20^ and it is well known that recovery of function remains difficult in certain areas such as episodic memory.^23^

Fatigue is a key obstacle to care in populations with neurological disorders (e.g. De Groot *et al.*^32^); thus, the management of fatigue is usually a priority (e.g. Brode and Melamed^33^). Strategies include identification of trigger situations, monitoring early signs, prioritizing tasks, arranging breaks, etc. (e.g. Malley^34^). For Long COVID patients, positive effects of pacing on return to work^35^ and quality of life^36^ have been observed in several longitudinal studies. In a study of 57 patients addressing the factors perpetuating fatigue (e.g. sleep-wake pattern, activity level, social support, worries regarding COVID-19, pain coping), cognitive-behavioural therapy (CBT) was also proven effective in reducing severe fatigue.^37^

Long COVID is also associated with psychological symptoms, including mood and anxiety disorders,^38-40^ which are known to affect cognitive performance (e.g. Dotson^41^). These symptoms may be explained by brain dysfunction,^42^ and the functional consequences of Long COVID. These symptoms may also increase the likelihood of problematic adaptive strategies, as in post-concussion or chronic pain contexts.^43^ Together, psychological and cognitive factors shape the overall quality of life.^42^ Thus, it is crucial to also consider psychological symptoms when developing treatment programmes for Long COVID.

Effective current practice in treating TBI often involves the use of CBT techniques,^44,45^ which have effects on anxiety and depression^46^ but also on behavioural disorders.^47^ Similarly, the application of CBT principles—such as stress management, behavioural activation, cognitive restructuring and goal setting—has been shown to be feasible and well accepted by Long COVID patients.^48^ While its effectiveness is still being studied (e.g. Koller *et al.*^49^), early findings are promising. Results on depression, anxiety and overall quality of life have been reported (in a case study^50^ and group studies^51,52^). CBT may also have a positive impact on the cognitive sphere. For instance, in a randomized controlled trial (RCT) with 34 Long COVID patients, Hausswirth *et al*.^53^ found that ten 30-min sessions of meditation significantly improved symptoms of depression and anxiety, sleep quality, physical and mental fatigue, as well as cognitive performance (reaction time-based tasks) with medium to large effect sizes.

In summary, since the first observations of persistent, disabling cognitive problems following SARS-CoV-2 infection, a variety of different interventions have been developed, in line with the usual practices in TBI patients. The results are promising for both cognitive and affective approaches using CBT techniques.^26,27,30,31^ However, there is no evidence on which is the best approach to propose. In this context, the COVCOG randomized controlled trial aimed to identify the most beneficial approach for Long COVID patients experiencing cognitive issues in daily life. For this purpose, we developed two brief, multidimensional psychoeducation and counselling interventions, targeting either (i) cognition and cognitive fatigue management using classical neuropsychological methods (cognitive arm), or (ii) stress and uncertainty management, behavioural activation, and goal setting using different CBT and relaxation techniques (affective arm). Their compared efficacy was tested in 130 Long COVID patients. We first sought to compare the effectiveness of the two programmes for perceived cognitive difficulties at 2 months post-intervention (primary outcomes). Given that the primary outcome concerned perceived cognitive difficulties and that the cognitive intervention directly addressed these difficulties in daily life, patients in the cognitive group were expected to improve more than those in the affective group. Then, we explored maintenance effects at an 8-month follow-up. Secondly, at both follow-up points, we aimed to compare the effects of the interventions on objective cognitive problems (in the areas of memory, attention and executive functions) and on self-reported measures of fatigue, sleep problems, psychological distress, quality of life, and work and activity impairments.

## Materials and methods

### General procedure

This randomized clinical trial (RCT) was conducted in accordance with the pre-registered protocol (ClinicalTrials.gov: NCT05167266). It involved a parallel-group design (allocation ratio 1:1), comparing two psychoeducational interventions targeting either cognitive (*N* = 65) or affective (*N* = 65) difficulties. Randomization with minimization was used to reduce heterogeneity between intervention groups. Detailed methodology (e.g. sample size calculation, minimization process and blinding process) can be found in the published protocol.^54^

At baseline (T0), and then 2 months (T1) and 8 months (T2) post-intervention, participants underwent a two-session assessment (90 min each) of attention, memory and executive functions (for more details, see Table 1). In addition, participants completed a set of questionnaires addressing cognitive complaints, fatigue, sleep difficulties, psychological distress, quality of life and impact of the difficulties on daily activities (see Table 2). Consistent with common clinical practice and following advice from patient partners, the assessment was divided into two sessions to minimize participant fatigue.

**Table 1:** Domains and functions measured in the neuropsychological assessment with selected tests and indices to cover the main cognitive domains

Domains	Functions	Test	Indices selected
Memory	Episodic verbal	Word list of the Repeatable Battery for the Assessment of Neuropsychological Status (RBANS)^55^	Immediate and delayed recall scores
	Episodic visuospatial	Brief Visuospatial Memory Test (BVMT-Revised)^56^	Immediate and delayed recall scores
Attention	Selective attention	Test of Attentional Performance (TAP)^57^; D2-R^58^	Response time and accuracy
	Divided attention	TAP^57^	Response time and accuracy
	Processing speed	TAP^57^ (i.e. response time at selective attention and divided attention tasks); STROOP test (naming and reading conditions)^59^; D2-R^58^	Response time
	Attentional fluctuation	^a^TAP^57^	Standard deviation scores
Executive functions	Inhibition	STROOP test^59^ (interference condition)	Response time, accuracy and interference index (interference RT—naming RT)
	Flexibility	Flexibility task of the TAP^57^	Response time and accuracy
	Working memory	Updating task of the TAP^57^; BROWN-PETERSON test^60^	Response time (updating) and accuracy (Brown-Peterson)
Language		Phonetic and semantic fluency^59^	Total number of words produced
Cognitive screening tool		MoCA^61^	Total score

^a^The tasks considered for this function are all the TAP tasks referenced in this table.

**Table 2:** Self-report questionnaires with selected scores and subscales, direction for interpretation and ranges for raw scores

	Questionnaire	Scores and subscales	Range
Executive and attention functioning complaints	Behaviour Rating Inventory of Executive Function (BRIEF-A)^62^	Total score (Global Executive Composite, GEC; primary outcome); Behavioural Regulation index (BRI); and Metacognition index (MI) subscales^a^	70–210
Memory functioning complaints	Multifactorial Memory Questionnaire (MMQ)^63^	Global composite score (primary outcome) and Satisfaction, Ability and Strategies subscales^b^	0–228
Fatigue	21-item Modified Fatigue Impact Scale (MFIS-21)^64^	Total score and three subscales: physical, cognitive and psychosocial fatigue^a^	0–84
Sleep problems	Pittsburgh Sleep Quality Inventory (PSQI)^65^	Total score^a^	0–21
Psychological distress	Outcome Questionnaire 45 (OQ-45)^66^	Total score and three subscales: symptom distress, interpersonal relations and social role^a^	0–180
Quality of life	Quality of Life Systemic Inventory (QLSI)^67^	Total score^a^	0–100
	EQ5D-5L^68^	VAS score^b^	0–100
Impact on work and activity	Work Productivity and Activity Impairment (WPAI)^69^	Two scores: work (absenteeism and presenteeism) and activity impairment^a^	0–100

^a^Higher scores indicate greater impairment.

^b^Lower scores indicate greater impairment.

This RCT was conducted and reported in accordance with the CONSORT-SPI 2018 guidelines for social and psychological intervention trials.

#### Patient partnership

The content of both interventions was developed in collaboration with Long COVID patients, incorporating their feedback during the early stages and throughout the process. Evaluation sessions were also tested with Long COVID patient partners to assess feasibility and make necessary adjustments before recruitment began. Patient partners were not themselves enrolled as study participants.

### Study setting, timeline and ethics

This trial was carried out in the context of a call from the Belgian Health Care Knowledge Centre (KCE). It was conducted by the University of Liège (Belgium) as a multicentre study at the University of Liège (the university hospital, CHU-Liège and the Psychology Clinic of University of Liège, CPLU), the university hospital of the Université Libre de Bruxelles (H.U.B.-ERASME hospital), the regional hospital of Liège (CHR-Liège) and the Mont Legia hospital of Liège (CHC-Liège). Patient enrolment and randomization were carried out between March 2022 and August 2023. Short-term follow-up evaluations (2 months post-intervention) took place between July 2022 and January 2024. Long-term follow-up evaluations (8 months post-intervention) took place between January 2023 and June 2024.

The study was approved by the Hospital-Faculty Ethics Committee of CHU-Liège (Belgium) under the reference number 2021/432. All participants gave written informed consent.

### Patients

The majority of patients (over 60%) were recruited through advertisements and via the routine-care pathways (over 35%). Participants were included if they were aged between 18 and 70, reported cognitive complaints that place the person in the top 20% of dissatisfied functioning on the BRIEF-A (Behaviour Rating Inventory of Executive Function) or MMQ (Multifactorial Memory Questionnaire) questionnaires, they have poor objective performance (supported by a score below the 20th percentile on at least one task of the cognitive battery) and had experienced at least one SARS-CoV-2 infection at least 3 months prior to their inclusion in the study confirmed by a PCR, antigen test or by a healthcare professional. Individuals with pre-existing neurological, cognitive or psychiatric disorders were excluded (for detailed criteria, see Willems *et al*.^54^).

All participants were offered a monetary compensation for journeys, which was divided into two parts and provided at different stages of the trial: once at the short-term follow-up and once at the long-term follow-up.

### Interventions

Both interventions involved four 90-min in-person sessions, 1 week apart. A 30-min reactivation session was administered according to the participant’s preferred mode (in-person, phone or videoconference) 1 month after the end of the intervention to discuss the current situation and any problems or questions that arose, and refresh some strategies. As this was a multicentre trial, the interventions were performed by different clinicians across different centres. However, all evaluations were conducted by a single blinded team (ULiège) at two centres (ERASME, ULiège). Manuals for both interventions were based on neuropsychological or CBT tools commonly used for the type of difficulties that Long COVID patients usually report and that have already been proven effective. Although the interventions incorporated tools from different approaches (e.g. cognitive rehabilitation, CBT), we chose to refer to them as psychoeducative interventions, as their primary focus was to implement adequate internal representations of the difficulties and to increase adaptive strategies. Various techniques were proposed to patients within the sessions, but without intensive training. For example, the Goal Management Training typically requires several sessions for a fully structured training.^70^ Here, it was only presented during one of the sessions of the cognitive intervention, as brief exposure can already show benefits.

The clinician’s approach to each session was slightly adapted to accommodate the specific difficulties of each patient. The intervention sessions were conducted by psychologists and neuropsychologists who were specifically instructed to them.

#### Cognitive intervention

This psychoeducational intervention targets metacognition and focuses on compensatory strategies using external aids, internal strategies and environmental adjustments through four different modules, each of which concerns a specific cognitive domain: fatigue and sleep difficulties, working memory and attention, executive functions and long-term memory (for further details, see Willems *et al.*^54^ and Table 3; manual available at https://orbi.uliege.be/handle/2268/329493). Contents in sessions were intensified by videotherapy and exercises accessible from home.

**Table 3:** Contents and key strategies of psychoeducational interventions

Cognitive intervention				
Contents	COVID-19 and cognition + Fatigue and sleep	Working memory and attentional functions	Executive functions	Long-term memory
Key strategies provided if relevant	General psychoeducation on Long COVID and cognitive difficulties; sleep and fatigue diary; sleep hygiene; fatigue level indicator and warning signs; planning, alternating and prioritizing activities according to the associated fatigue; pacing and breaks^70^	Environmental adjustments to reduce interference; strategies to reduce pressure on short-term memory and attention (e.g. reduce dual task situations); time pressure management (TPM) for slowed information processing^71^	Problem identification; planning and monitoring performance; Goal Management Training^72^	Internal strategies; external memory compensations and aids; errorless and spaced retrieval; reminiscence; distributed learning; retrieval techniques^21^

Affective intervention				
Contents	Recognizing difficulties and affective states	Tolerating uncertainty and worries associated with difficulties	Accepting and communicating emotions and difficulties	Behavioural activation
Key strategies provided if relevant	Identification of dysregulatory thought-patterns and reactions; functional analysis; Stimulus–Organism–Response–Consequence (SORC) model^73^	Management of intolerance of uncertainty; beliefs about worry; poor problem orientation and cognitive avoidance^74^	Self-observation of emotions; assertive and effective communication principles (Fanget and Rouchouse’s principles)^75^	Identification of values to guide choices; impact of ruminations; construction of a behavioural activation plan^76^

#### Affective intervention

This psychoeducational intervention targets self-efficacy for emotion management and regulation of behaviours impacting the perception of difficulties in daily living activities (see Willems *et al.*^54^ and Table 3, manual available at https://hdl.handle.net/2268/329180). Each session included body awareness and relaxation exercises and was intensified by notes and home exercises.

### Primary and secondary outcomes

Assessments were conducted at three time points: baseline (T0), 2 months follow-up (T1) and 8 months follow-up (T2). Primary outcomes were cognitive complaints at T1. Secondary outcomes included all other measures assessed at T1 and T2, as well as cognitive complaints at T2.

#### Primary outcomes

The primary outcomes were cognitive complaints measured at 2 months (T1) using two questionnaires (see Table 2), one of which assessed executive control (BRIEF-A) and the other memory difficulties (MMQ). The questionnaires were administered twice at each time point (T0, T1 and T2) via Castor Electronic Data Capture (https://www.castoredc.com/) over a 2-week interval to minimize the effects of occasional fluctuations in difficulties. Analyses of cognitive complaints were carried out on *Z*-scores based on population norms released with the task, with lower scores indicating higher difficulties. For the BRIEF-A, a mean *Z*-score of the Global Executive Composite was calculated from both administrations. For the MMQ, a *Z*-score for each subscale based on the norm values was first computed, and these *Z*-scores were then averaged, yielding a single *Z*-score per administration. A final mean *Z*-score from both administrations was used in the analyses.

#### Secondary outcomes

The subscales of both cognitive complaint questionnaires were analysed at 2- and 8-month follow-up (T1, T2). Secondary outcomes also included cognitive performance (see Table 1) at T1 and T2 (patient’s worst *Z*-score computed on overpopulation norms). Classical neuropsychological asks covering the main cognitive domains (attention, working memory, long-term memory and executive functions) were used: RBANS (Repeatable Battery for the Assessment of Neuropsychological Status), BVMT-R (Brief Visuospatial Memory Test Revised), TAP (Test of Attentional Performance), D2-R, Stroop, Brown-Peterson and fluency tasks (see Table 1 for more details and references). Alternate forms were used for each administration for the memory tasks (RBANS and BVMT-R). Other secondary outcomes included fatigue (Modified Fatigue Impact Scale; MFIS), sleep difficulties (Pittsburgh Sleep Quality Index; PSQI), psychological distress (Outcome questionnaire 45; OQ-45), quality of life (Quality of Life Systemic Inventory; QLSI, and EQ-5D-5L) and work productivity (Work Productivity and Activity Impairment; WPAI) at T1 and T2 (raw scores; see Table 2).

### Blinding procedure

Obviously, clinicians delivering the interventions and patients were not blinded to the intervention; however, the expected benefits of both interventions were emphasized. The neuropsychologists conducting the cognitive assessments and the statistician performing the statistical analyses were blinded to group assignment. Before starting each session, neuropsychologists reminded the participants that they could not give any information allowing them to know which intervention they have followed. All data analyses were conducted on interventions labelled ‘A’ and ‘B’, without any indication of which was the cognitive or affective one. Finally, another non-blinded psychologist was responsible for the randomization process and for interactions with participants and intervention clinicians when necessary.

### Adherence and satisfaction

Adherence and satisfaction were evaluated for clinicians and patients based on the percentage of patients who actually completed the intervention, the number of prescribed exercises performed, Likert scales (0–10) measuring the perceived usefulness of each intervention before randomization, credibility and expectation at the first intervention session, and satisfaction and perceived feasibility at the 2-month follow-up.

### Statistical analyses

Analyses to verify the comparability of the two intervention groups’ demographic and clinical characteristics were conducted using *t*-tests or Fisher tests. The effects of the intervention group on the primary outcome measures (BRIEF-A and MMQ scores) were analysed using linear mixed models (LMMs) with the time of the measure (T0 and T1), the intervention (nested within the centres), and the time-by-intervention interaction as fixed factors. Centres and participants were considered random factors. To account for variables that could potentially affect intervention response, age (years), education level (primary or vocational secondary education; general secondary education; higher education—bachelor level; higher education—master level or above), sex and the extent of the objective cognitive deficit (worst score among the worst *Z*-scores per cognitive domain) were also included in the model as stratification factors. The 95% confidence interval (CI95) and standardized effect sizes (SES) were calculated, and SES were interpreted as follows: 0.2–0.49 small, 0.5–0.79 moderate and ≥0.8 large effect size.^77^

Similar analyses were conducted on secondary outcome measures—the patient’s worst *Z*-score for each cognitive domain separately and (sub)score on each questionnaire. Additional LMM included the three time-points (T0, T1 and T2) to assess the maintenance of effects across time. Although LMMs are generally robust to slight deviations from their assumptions, we systematically verified the conditions for the application of LMM normality of residuals, homoscedasticity and linearity.

Objective cognitive impairment was measured using the worst *Z*-score per cognitive domain rather than computing a composite score per domain. This choice was motivated by the fact that individuals suffering from Long COVID have very heterogeneous disorders and that a patient may present with a specific impairment (e.g. divided attention) that substantially affects daily functioning, even if performance on other tasks within the same domain is preserved. Averaging across measures would mask such isolated but clinically meaningful deficits and would mainly allow for detecting those patients with more generalized impairments, which would not align with our aim of identifying clinically relevant difficulties.

Primary outcomes resulting from the short-term follow-up (T1) were first analysed using an intention-to-treat (ITT) approach, meaning that participants were considered in their originally assigned groups, regardless of compliance. To account for missing information due to patient dropout, we relied on the LMM and considered the baseline data as part of the outcome matrix. Secondly, all data were analysed with a per-protocol (PP) approach, removing data from dropouts. Additional analyses for the long-term follow-up (T2) were conducted using only a PP approach.

Reliable change indices between T0 and T1 were calculated for each participant on the primary outcome measures: global measures of executive complaints (GEC score at BRIEF-A) and memory complaints (three MMQ subscales). For this, a CI95 was calculated on the T0 score using the standard error of measurement. A significant change was considered to exist when the T1 score fell outside this CI.

Statistical analyses were conducted with R statistical software (version 4.1)^78^ using a script generated for these analyses. Due to the Bonferroni correction, a statistical threshold of *P* < 0.025 was used for primary outcome measures, whereas for secondary measures, we adopted an exploratory, hypothesis-generating approach to preserve statistical power with a threshold of *P* < 0.05. Analyses of cognitive complaints and objective performances were carried out on *Z*-scores based on population norms, with lower scores indicating greater difficulties. Analyses of somatic and psychological questionnaire results were conducted using raw scores. Adherence and satisfaction for patients were analysed using *t*-tests on the scores of the Likert scales. Details on the sample size calculation and power analysis are provided in the pre-registered protocol.^54^

## Results

### Sample description, attrition and group equivalence

At T0, 139 Long COVID patients underwent neuropsychological assessment. Five patients did not meet the inclusion/exclusion criteria, one patient was excluded because of non-credible test performances and three patients withdrew from the study after the first visit, resulting in 130 patients randomized (*N* = 65 cognitive and *N* = 65 affective intervention). In total, 114 patients completed T1 (118 with primary outcomes), and 102 completed T2 (108 with primary outcomes).

**Figure 1:**
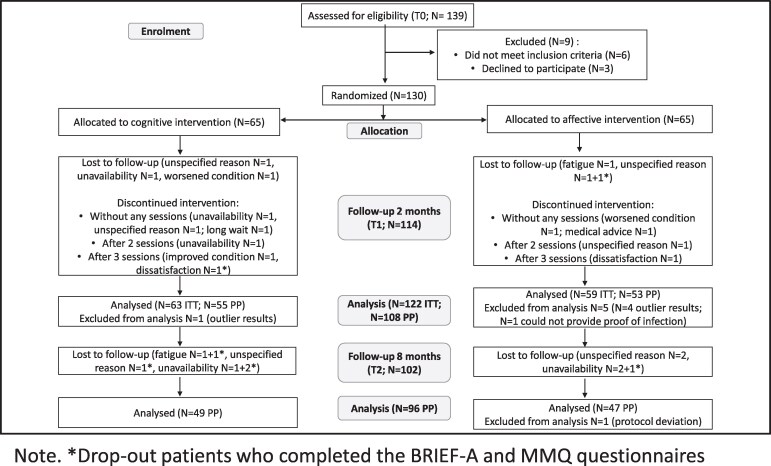
**Participant’s flow diagram.** Flow diagram illustrating the participants’ process through the phases of the trial.

Eight patients were removed from the statistical analyses: seven due to outlier results at the baseline evaluation (identification of multivariate outliers using the Mahalanobis distance^79,80^ and critical *X*² value; who showed clinically not credible cognitive scores), and one patient was incorrectly randomized (could not provide proof of infection). Thus, 122 patients were analysed according to ITT principles (63 cognitive, 59 affective), and 108 according to the PP approach (53 cognitive and 55 affective). Statistical analyses of outcomes 8 months post-intervention were conducted on 96 patients with the PP approach (49 cognitive, 47 affective). Figure 1 illustrates participant flow through the trial.

Outcomes from the baseline evaluation were analysed in a previous study.^11^

The 122 patients had a mean age of 47 years and a mean of 14 years of education; 70% were women (see Supplementary Table 1 for further details based on ITT principles and Supplementary Table 2 for PP details). Most participants (88.5%) had had mild to moderate SARS-CoV-2 infections (see Supplementary Table 1 for detailed severity criteria), averaging 1.7 infection episodes, of which at least one was medically certified. Their infection(s) occurred between March 2020 and November 2023, and the mean interval between the first infection and study inclusion was 20.9 months. The patients’ most common pre-existing conditions, as well as their sociodemographic characteristics and COVID-19 history, are described in Supplementary Table 1. Prior to their SARS-CoV-2 infection, 93.4% worked or studied in higher education, and only 1.6% were on sick leave. At study inclusion, 59.8% worked or studied in higher education, and 33.6% were on sick leave.

Baseline data were analysed and discussed in a previous study.^11^ There were no differences between groups for demographic characteristics (sex, age, years of education, occupation at study inclusion; all *P*s > 0.34; see Table 4) or delay between first infection and study inclusion (*Z* = 1.87, *P* = 0.07). No differences were found at T0 for subjective cognitive complaints (BRIEF-A and MMQ scores), objective cognitive performance (patients’ worst *Z*-score) or number of patients with at least one task below the clinical thresholds (P5 and P2) (all *P*s > 0.3). Self-reported fatigue and psychological distress were also similar between groups (all *P*s > 0.23).

**Table 4:** Comparison between intervention groups at baseline on demographics and clinical characteristics

	Affective intervention (*n* = 59)	Cognitive intervention (*n* = 63)	*t* or *X*^2^	*P*
Age (years; mean (SD))	46.7 (10.5)	47.3 (9.8)	−0.31	0.75
Female (n, %)	42 (71.2%)	43 (68.3%)	0.12	0.73
Education (years; mean (SD))	13.9 (3)	14.2 (3)	−0.55	0.58
Delay between infection and study inclusion [months; mean (SD)]	19.1 (8.6)	22.6 (8.4)	1.87	0.07
Subjective cognitive complaints [BRIEF-A; mean (SD)]	137.4 (21.7)	139 (20.6)	−0.44	0.66
Subjective cognitive complaints [MMQ; mean (SD)]	94.2 (20.1)	94.3 (17)	0.06	0.95
Objective cognitive performance (patient’s worst *Z*-score) [mean (SD)]	−1.49 (0.7)	−1.54 (0.8)	0.38	0.71
Pre-randomization perceived usefulness [mean (SD)]	7.3 (2.1)	8.6 (1.4)	6.04	<0.001
Credibility and expectations at first session [mean (SD)]	7.7 (1.3)	8.2 (1)	2.23	0.03
Satisfaction at end of intervention [mean (SD)]	8 (1.6)	7.9 (1.8)	−0.57	0.57
Perceived feasibility at end of intervention [mean (SD)]	7.4 (1.8)	6.7 (2)	−2.03	0.045
Fatigue [MFIS; mean (SD)]	61.9 (13.8)	63.5 (12.6)	−0.65	0.52
Psychological distress [OQ-45; mean (SD)]	67.4 (22.4)	72.5 (23.7)	−1.22	0.23
Patients with objective cognitive deficit (with at least one task < P5) [*n* (%)]	25 (42.4%)	25 (39.7%)	0.09	0.76
Patients with objective cognitive deficit (with at least one task < P2) [*n* (%)]	11 (18.6%)	16 (25.4%)	0.81	0.37
Occupation at study inclusion: Actively employed/sick leave (*n*)	44/15	42/21	0.92	0.34

#### Compliance and deviations

Of the 130 randomized patients, 125 (96.2%) participated in the rehabilitation programmes (62/65 cognitive, 63/65 affective); 120 of 125 patients completed all sessions (59 cognitive, 61 affective); 3 patients completed 3 of the 4 sessions and 2 completed 2 sessions. 118 patients completed the reactivation session. The majority of patients performed all the home exercises prescribed (115 of 125). Only minor protocol deviations were noted (e.g. one rather than two BRIEF-A and MMQ administrations, or variations in follow-up timing; see Supplementary Information 1).

No harms, unintended effects or adverse events related to the intervention were observed or reported during the trial.

### Primary outcomes

#### Short-term effects on cognitive complaints

The PP and ITT models yielded equivalent results; the following results describe the ITT analyses (see Table 5; for the PP models, see Supplementary Table 3). Effect sizes of the difference between the two intervention groups at T1 ranged from −0.36 to 0.5.

**Table 5:** Raw scores of LMM on cognitive complaints (primary outcomes) at 2-month follow-up following an ITT approach

	Time effect		Affective intervention				Cognitive intervention				Time-by-group interaction			
	*F*	*P*	Baseline (mean ± SD)	2 months post-intervention (mean ± SD)	*d* (time effect)	CI95	Baseline (mean ± SD)	2 months post-intervention (mean ± SD)	*d* (time effect)	CI95	*d* (interaction effect)	CI95	*F*	*P*
BRIEF (GEC)	17.4	<0.001	137.4 (±21.7)	132.5 (±27.3)	0.59	[0.22, 0.96]	139 (±20.6)	134.1 (±20.7)	0.48	[0.13, 0.84]	0.02	[−0.08, 0.12]	0.2	0.68
MMQ (composite score)	16.3	<0.001	94.2 (±20.1)	100.2 (±23)	0.54	[0.17, 0.91]	94.3 (±17)	103.4 (±19.3)	0.49	[0.14, 0.85]	0.001	[−0.12, 0.14]	0.03	0.85

GEC, global executive composite.

**Figure 2:**
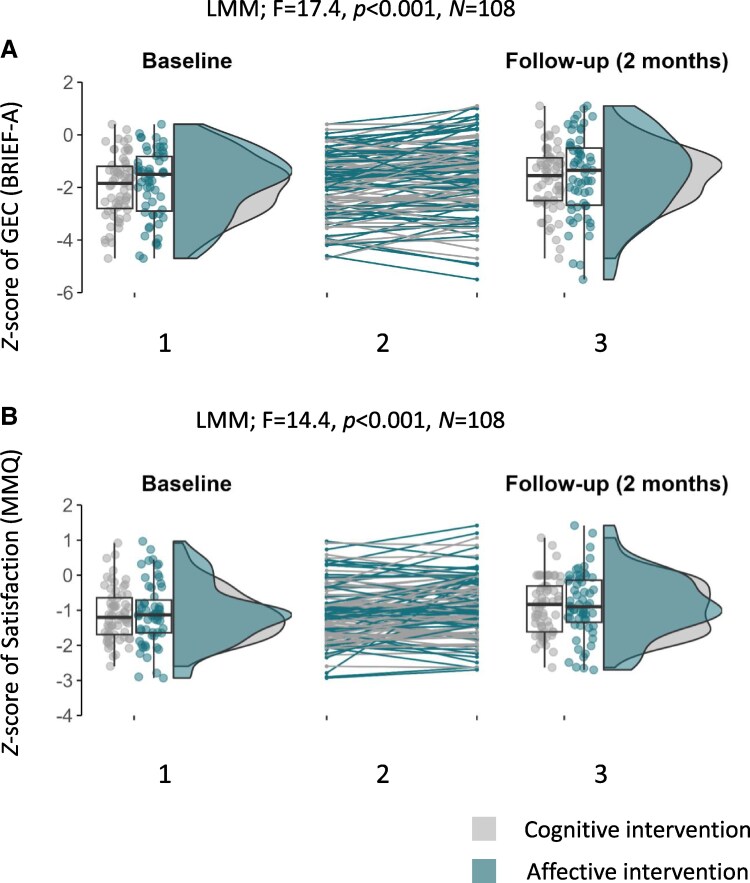
**Executive/attentional complaints and memory dissatisfaction at T0 and T1.** (**A**) Executive and attentional complaints measured with the BRIEF-A (GEC *Z*-score) at T0 (A.1) and T1 (A.3). (**B**) Dissatisfaction with memory measured with the MMQ (satisfaction subscale *Z*-score) at T0 (B.1) and T1 (B.3). The trajectories between T0 and T1 for each patient are represented in the central sections (A.2 and B.2). The *y*-axes represent the severity of complaints: the lower the score, the more complaints.

For the global BRIEF-A score, LMM analysis revealed a significant decrease in complaints over time (*F* = 17.4, *P* < 0.001; see Fig. 2A), with small to moderate time effect sizes for both groups (cognitive: *d* = 0.48, *P* = 0.008; affective: *d* = 0.59, *P* = 0.002), but no time-by-group interaction, indicating that trajectories did not differ between arms. These findings were substantively similar for the MMQ composite score, with a significant improvement over time (*F* = 16.33, *P* < 0.001), and moderate time effect sizes for both groups (cognitive: *d* = 0.49, *P* = 0.007; affective: *d* = 0.54, *P* = 0.004), but no interaction. Statistically significant time effects identified by the LMMs for both primary and secondary outcomes under a PP approach are summarized in Supplementary Table 12.

Reliable change revealed an improvement in 23 patients (37%) in the cognitive group and 23 (39%) in the affective group for the BRIEF global score. An improvement in at least one of the MMQ subtests was observed for 22 patients (35%) in the cognitive group and 21 (36%) in the affective group.

### Secondary outcomes

#### Long-term effects on cognitive complaints

LMM, including the three time-points on the BRIEF-A and MMQ scores, showed that the significant improvement is maintained at T2, with no difference between T1 and T2 (all *P*s > 0.55; see Table 6). However, only the cognitive group showed a moderate effect of time for BRIEF-A at T2 in comparison with T0 (cognitive: *d* = 0.55, *P* = 0.006; affective: *d* = 0.38, *P* = 0.1). Moreover, the effect on the MMQ was large in the cognitive arm (*d* = 0.85, *P* < 0.001) but moderate in the affective arm (*d* = 0.54, *P* = 0.01).

**Table 6:** Results of LMM on cognitive complaints at 8-month follow-up following a PP approach

	Scores at T2		Statistical values for comparison T2 versus T0				Statistical values for comparison T2 versus T1			
	Affective intervention	Cognitive intervention	Affective intervention		Cognitive intervention		Affective intervention		Cognitive intervention	
	Mean (±SD)	Mean (±SD)	*d*	CI95	*d*	CI95	*d*	IC95	*d*	CI95
BRIEF (GEC)	133.9 (±28.3)	134.6 (±23.5)	0.38	**[0.02, 0.74]**	**0.55**	[0.2, 0.91]	−0.19	[−0.56, 0.17]	0.08	[−0.27, 0.43]
BRIEF BRI	53.6 (±12.8)	55.4 (±10.7)	0.31	[−0.09, 0.71]	0.62	[0.23, 1]	−0.1	[−0.5, 0.29]	0.2	[−0.19, 0.58]
BRIEF MI	80.3 (±17.4)	79.2 (±15)	0.42	[0.02, 0.82]	0.59	[0.2, 0.97]	−0.13	[−0.52, 0.27]	0.18	[−0.2, 0.57]
MMQ composite score	99.8 (±25)	105.2 (20.8)	**0.54**	**[0.18, 0.91]**	**0.85**	**[0.5, 1.2]**	−0.06	[−0.42, 0.3]	0.05	[−0.31, 0.4]
MMQ satisfaction	24.5 (±15.4)	24.5 (±13.3)	0.44	[0.04, 0.83]	0.84	[0.45, 1.23]	0.04	[−0.44, 0.35]	0.30	[0.08, 0.68]
MMQ ability	34.3 (±14.3)	37.9 (±13)	0.56	[0.16, 0.96]	0.88	[0.49, 1.27]	0.09	[−0.48, 0.31]	0.14	[−0.24, 0.53]
MMQ strategies	40.9 (±11.7)	42.8 (±10.5)	0.008	[−0.38, 0.40]	0.08	[−0.3, 0.46]	0.03	[−0.37, 0.42]	0.41	[−0.8, −0.03]

GEC, Global Executive Composite; BRI, Behavioural Regulation Index; MI, Metacognition Index.

Significant effects (*P* < 0.05) are marked in bold. T0: baseline; T1: 2-month follow-up; T2: 8-month follow-up.

#### Short- and long-term effects on specific cognitive complaints

Regarding the cognitive questionnaires’ subscales, similar time effects were noted for the BRIEF-A metacognition and behavioural regulation indices, and for the MMQ satisfaction (see Fig. 2B) and ability subscales, with moderate time effects in both groups, but no group or interaction effect (all *P*s > 0.12; see Supplementary Table 4). These moderate effects of time persisted at T2 (see Table 6). However, there was no effect of time on the MMQ strategies subscale, at either T1 or T2 (all *P*s > 0.5).

#### Short- and long-term effects on somatic and functional complaints

Descriptive statistics for questionnaire scores are reported in Supplementary Tables 5 (ITT at short-term and PP at long-term follow-up) and 6 (PP approach at both follow-ups). The results of LMM on the somatic complaints questionnaires are included in Supplementary Table 4. Long-term effects are included in Supplementary Table 7.

##### Fatigue and sleep

LMM revealed a time effect between T0 and T1 on the MFIS global score (*F* = 20, *P* < 0.001) with moderate effects in both arms (cognitive: *d* = 0.54, *P* = 0.003; affective: *d* = 0.6, *P* = 0.001), again without an interaction effect. A moderate time effect was also found for the physical and cognitive fatigue subscales, with both arms evolving similarly (all *P*s < 0.001). In contrast, for the psychosocial fatigue subscale, we observed no effect of time, but a significant interaction (*F* = 4.24, *P* = 0.04) with a moderate improvement only in the cognitive group (cognitive: *d* = 0.48 *P* = 0.008; affective: *d* = −0.05, *P* = 0.79). Similar analyses, including long-term follow-up, showed that the time effect was maintained at 8 months, with no difference between T1 and T2 (all *P*s > 0.5), and a significant time effect between T0 and T2 (all *P*s < 0.046).

LMM on the PSQI global score measuring sleep difficulties revealed no interaction. A time effect was observed (*F* = 5.43, *P* = 0.02), mainly due to the cognitive group (*d* = 0.38, *P* = 0.04); there was no significant effect for the affective group (*d* = 0.22; *P* = 0.23). A sex effect was observed (*F* = 8.87, *P* = 0.004): men had more complaints (women mean = 8, SD = 3.4; men mean = 9.2, SD = 4.3). At the long-term follow-up, the time effect was no longer observed. A sex effect was noted between the baseline and the 8-month follow-up (*F* = 5.96; *P* = 0.016), with women reporting more complaints (women mean = 8.2, SD = 3.7; men mean = 6.5, SD = 4.4).

##### Psychological distress and quality of life

LMM showed no significant effect on the OQ-45 global score (all *P*s > 0.055). As for the subscales, a time effect was noted for symptom distress (*F* = 7.23, *P* = 0.008), explained by a moderate effect for the cognitive group (*d* = 0.51, *P* = 0.005) but no effect for the affective group (*d* = 0.18, *P* = 0.3). An interaction effect was observed for interpersonal relations (*F* = 9.98, *P* = 0.002), with a significantly small effect, but statistically significant only for the affective group (affective: *d* = 0.45, *P* = 0.01; cognitive: *d* = −0.35, *P* = 0.05). There was also a sex effect (*F* = 5.09, *P* = 0.03) with men reporting greater distress (women mean = 11.7, SD = 6; men mean = 12, SD = 6.8). No significant effect was observed on the social role subscale (all *P*s > 0.36). At the long-term follow-up, the time effect for symptom distress was maintained, with no difference between the 2- and 8-month follow-ups (all *P*s > 0.74), and a significant time effect between T0 and T2 (*F* = 5.24, *P* = 0.006). The effect on interpersonal relations was not maintained over time (cognitive: *d* = 0.09, *P* = 0.87; affective: *d* = −0.27, *P* = 0.3).

LMM showed no interaction effect on the two scales measuring quality of life (QLSI and EQ-5D-5L). However, a significant time effect was observed on the QLSI (*F* = 12.87, *P* < 0.001), with small to moderate effects for both groups (cognitive: *d* = 0.54, *P* = 0.003; affective: *d* = 0.39, *P* = 0.04). Again, we noted a sex effect (*F* = 4.05, *P* = 0.046) with men reporting a lower quality of life (women mean = 7.4, SD = 7.2; men mean = 9.9, SD = 7.7). At T2, a time effect was also noted in comparison to T0 (*F* = 5.33, *P* = 0.005).

For the VAS score at the EQ-5D-5L, a time effect was also observed (*F* = 10.26, *P* = 0.002), mainly explained by a moderate effect in the cognitive group (cognitive: *d* = 0.59, *P* = 0.001; affective: *d* = 0.22, *P* = 0.22). At T2, the quality of life was higher than at T0 (*F* = 7.54, *P* < 0.001), but there was no difference from T1 (all *P*s > 0.88).

##### Work and activity

LMM revealed no interaction effect on the WPAI (all *P*s > 0.45). A time effect for the measure of impact on work was observed (*F* = 7.58, *P* = 0.007), mainly explained by a moderate effect in the affective group (affective: *d* = 0.57, *P* = 0.01; cognitive: *d* = 0.33, *P* = 0.2). No effect was observed for impact on daily activities (all *P*s > 0.07). At T2, the time effect on overall work impairment (absenteeism and presenteeism) was maintained for both the affective group (*F* = 9.43, *P* < 0.001), with a moderate effect size (*d* = 0.73, *P* = 0.004), and the cognitive group, also with a moderate effect size (*d* = 0.68, *P* = 0.013).

#### Short- and long-term effects on cognitive performance

The mean MoCA score remained at 27 across time points. Below-cut-off scores (<26) were observed in 19 patients (15.4%) at T0, 25 at T1 (21% ITT; 22 patients, 20.4% PP) and 13 at T2 (12%).

As for the cognitive assessment, the mean scores at baseline can be found in Supplementary Table 8. LMM on patients’ worst scores at T1 (see Supplementary Table 9) showed no interaction effects (all *P*s > 0.18). Small to moderate time effects on attention, working memory and long-term memory were observed (all *P*s < 0.002). Attention improved significantly only in the cognitive arm (cognitive: *d* = 0.57, *P* = 0.002; affective: *d* = 0.24, *P* = 0.21), and the same was true of long-term memory (cognitive: *d* = 0.68, *P* = 0.0002; affective: *d* = 0.33, *P* = 0.075). Improvements in working memory were observed only in the affective arm (affective: *d* = 0.56, *P* = 0.003; cognitive: *d* = 0.26, *P* = 0.15). No effect was observed for the worst score in executive functions (all *P*s > 0.055).

At T2, effects were maintained over time (all *P*s > 0.18). In the cognitive group, worst scores improved from T0 to T2 in attention (*P* < 0.001), long-term memory (*P* = 0.001) and working memory (*P* = 0.007). There was no improvement in the affective group (*P* = 0.05, *P* = 0.49 and *P* = 0.07, respectively; see Supplementary Table 10). No effect was found for executive functions (all *P*s > 0.2).

### Expectations and satisfaction measures

Among patients who completed a final questionnaire on the content of the intervention (*N* = 55), mean satisfaction was 7.7 of 10 (±1.6; range: 2–10). Perceived usefulness at pre-randomization and credibility and expectations after the first session were higher for the cognitive intervention (see Table 4). However, patients in both groups were equally satisfied at the end of the interventions, even though the cognitive intervention was perceived as less feasible than the affective one.

## Discussion

This RCT aimed to determine the most beneficial approach to manage cognitive problems in Long COVID, using a randomized parallel two-group design, in which 122 Long COVID patients underwent neuropsychological evaluations 2 and 8 months after participating in a psychoeducational intervention. The intervention focused on either metacognition and management of mental fatigue (cognitive arm) or stress management, behavioural activation, cognitive restructuring and goal setting (affective arm). The cognitive intervention was expected to produce better effects, as it directly addressed cognitive complaints, unlike the intervention targeting affective dimensions. To our knowledge, this is the first study to directly compare the effectiveness of these two approaches.

### Feasibility and satisfaction

The low dropout rate and high adherence confirm the feasibility of both interventions: 92% of patients attended all sessions, and 96% completed all the prescribed exercises. Although the cognitive intervention was rated as more useful and credible prior to randomization, post-intervention satisfaction was positive and similar in both arms.

### Effects of psychoeducative interventions

We found that Long COVID patients showed a decrease in executive and attention (assessed with the BRIEF-A), and memory complaints (assessed with the MMQ) after only 6 h of psychoeducation. Furthermore, reliable change indices between baseline and the 2-month follow-up showed that over a third of patients improved beyond the changes that could be expected due to measurement error or natural variability. However, contrary to expectations, the cognitive intervention was not observed to have superior effects for these primary outcomes.

Improvements were also observed in fatigue, sleep, two subscales of psychological distress, quality of life and objective cognitive performance (attention, working memory and long-term memory) with small to moderate effect sizes. The cognitive intervention proved superior for some secondary outcomes: psychosocial fatigue, sleep, distress symptoms and cognitive performances (attention and long-term memory). Finally, results on the long-term follow-up (8 months post-intervention) showed that the improvements at 2 months persisted over time. A sex effect was observed for some measures at T1 (sleep problems, quality of life, interpersonal relations), where men reported more difficulties. This may be due to a sampling bias: there were fewer men in the sample, and those who volunteered may have been more distressed and not fully representative of the male Long COVID population. For sleep problems, men seem to improve more than women, since at T2, women reported more difficulties than men. Further studies should investigate this hypothesis with a more balanced sample.

For most outcomes, a time effect without a statistically significant group interaction was observed. Given that previous studies have shown a general trend towards a decrease in cognitive difficulties over time, for both subjective complaints (e.g. Badinlou *et al.*^81^) and objective measures,^82^ we cannot rule out a non-specific effect, independent of the intervention. Then, we carried out a posteriori linear regression analyses (see Supplementary Table 11) with time between first SARS-CoV-2 infection and study inclusion or short-term follow-up evaluation. Time elapsed since infection does not explain the changes in self-reported difficulties, either at baseline or 2 months post-intervention (BRIEF-A and MMQ scores; all *P*s > 0.07), making it highly unlikely that spontaneous recovery alone explains the observed improvements. However, the possible influence of broader, non-specific factors cannot be excluded and is further addressed in the limitations section of this study.

Another argument against spontaneous recovery is the improvements specific to each arm that could be explained by the contents of the interventional approach. For example, the statistically significant improvements in sleep difficulties and the psychosocial dimension of fatigue only in the cognitive arm are consistent with the presence of a module targeting these topics. These patients also demonstrated greater improvements in cognitive performances in attention, working memory and long-term memory. In contrast, the affective group showed greater improvements in the interpersonal dimension of fatigue, aligning with that arm’s emphasis on behavioural activation and improving communication with relatives. Those results may support the idea that the interventions have specific impacts beyond a placebo effect. Given the exploratory nature of the secondary outcomes, this hypothesis of intervention-specific effects should be further explored in future trials.

With regard to cognitive complaints, our findings support the value of psychoeducation approaches focusing on the management of cognitive functioning in daily activities or using CBT techniques to manage stress and increase goal-related activities.^27,28^ Indeed, CBT can make changes impacting quality of life and potentially reducing daily functioning issues and cognitive complaints.^83^

Regarding objective cognitive performance, in the cognitive arm, small to moderate improvements were noted after 2 months in attention and long-term memory, and after 8 months in working memory. Patients in the cognitive arm, therefore, appeared to better manage cognitive tasks, as previously observed (e.g. Braga *et al.*^26^). The affective group also showed an improvement in working memory at 2 months, possibly due to the reduction in anxio-depressive symptomatology, which is known to have a major impact on objective performance.^84^ A cognitive improvement has also been noted in other studies of affect-focused psychoeducation (e.g. Hausswirth *et al.*^53^). However, the effect was not maintained at 8 months, while measures of psychological distress remained stable.

In contrast, objective cognitive difficulties measured with the MoCA remained unchanged over time, highlighting the importance of conducting full neuropsychological assessments with instruments that are more sensitive than global brief screening tools not specifically designed for Long COVID patients.^85^

A slight improvement was observed after both interventions for the somatic complaints of fatigue (small to moderate effect) and sleep difficulties (small effect). A single session on management of fatigue and sleep in the cognitive arm could therefore already have positive effects. Consequently, increasing awareness of the first signs of fatigue and managing fatiguing activities is an approach that may be worthwhile. Communicating personal limits, as proposed in the affective arm, could also have positive effects. The two approaches could be combined to maximize their impact.

Positive time effects were also noted on symptom distress (small effect) and quality of life (small to moderate effect). Those results align with previous research into psychoeducational interventions in Long COVID that found benefits for mood^27,28^ and quality of life^86^ with similar effect sizes and reinforce the value of these particular approaches.

We also observed a time effect in both arms for work-related difficulties (small to moderate effects), though the affective arm had a faster effect (at 2 months versus at 8 months for the cognitive arm). One potential explanation could reside in the behavioural activation principles introduced in the affective arm, which seems to be a key ingredient of return-to-work-focused therapies.^87^

Those time effects on complaints were maintained over time (at 8 months post-intervention). One possible explanation is that the strategies taught may have been incorporated into patient’s daily routines, supporting a long-term management of difficulties.

However, the conclusions regarding the positive effects associated with both interventions should be viewed with caution. In addition to a lack of interaction effect between the two groups, we observed small to moderate effect sizes, and many patients remained below the clinical threshold for a large number of measures despite improvements—e.g. 85% still reported pathological fatigue, 79% sleep difficulties, 56% psychological distress and 64% low quality of life (see Supplementary Tables 5 and 6 for more details). These findings highlight that the observed improvements may not necessarily translate into clinically meaningful functional gains in patients’ daily lives, and that a brief intervention (6 h) may be insufficient for this clinical population. Thus, there remains substantial room for optimizing these interventions, for example, by adjusting their dosage or extending their duration. Further studies are therefore needed to explore the optimal length and content of intervention.

However, two conclusions can be drawn from these results. Firstly, these difficulties are so severe at baseline that, despite moderate improvements, they remain above clinical thresholds over time. The prevalence and severity here align with what is observed, for example, for fatigue, which appears to be the most common symptom in Long COVID patients^81,88,89,90^; sleep, which can get worse^81^; psychological symptoms^91^; and negative effects on the ability to work.^5,16,90,92^ Secondly, some patients potentially need additional intervention sessions.

## Future prospects

Our findings suggest that a combined intervention could generate benefits. Evidence-based treatments such as CBT and cognitive rehabilitation have been successfully combined for other conditions with chronic neuropsychiatric symptoms, such as post-concussion syndrome,^93,94^ fibromyalgia and chronic fatigue syndrome.^95^ In the context of Long COVID, rehabilitation programmes including psychoeducation on cognitive dysfunction, emotion regulation and behavioural activation strategies appear particularly relevant.^96^ In light of the literature and our results, a combination of both interventions’ modules seems appropriate for this population.

Additionally, in a previous study on baseline outcomes,^11^ we identified three different Long COVID patient profiles. This supports the notion that Long COVID represents a spectrum of clinical phenotypes rather than a uniform condition. Consistent with this, research on cluster distribution has identified distinct clinical profiles based on a variety of medical and demographic variables.^97,98^ Distinct cognitive profiles^99,100^ and psychological profiles^101,102^ had also been described. Such heterogeneity likely reflects multiple underlying mechanisms^103^ and may contribute to differential responses to interventions, underscoring the importance of developing tailored rehabilitation pathways. Furthermore, the existence of diverse phenotypes raises important questions about the long-term evolution of cognitive difficulties, including the potential increased risk of neurodegenerative disorders following SARS-CoV-2 infection, as suggested by recent studies^104^ and by evidence from other viral infections.^105^ Future interventional studies should therefore focus on the preventive effect on long-term progression.

### Strengths

The content of both interventions was discussed in the initial stages of the study and throughout the entire process in collaboration with Long COVID patients experiencing cognitive difficulties. Their feedback was integrated throughout the process, ensuring that the programmes addressed real patients’ needs. Patient involvement in research is increasingly recommended because of its positive impact on both patients (e.g. knowledge and skill development) and the research process (e.g. contribution of the ‘patient voice’ to research) (for a review, see Bird *et al.*^106^).

Another key strength is the longitudinal design, with two follow-up points in the short- and long-term (2 and 8 months post-intervention). Most studies on the efficacy of interventions in Long COVID patients focus on short-term effects,^27,86^ making it difficult to determine long-term benefits and patient trajectories.

## Limitations

The absence of a control group limits direct attribution of effects to the interventions. A no-treatment control condition was not included for ethical reasons, as it would have involved withholding intervention from individuals experiencing ongoing clinically significant cognitive symptoms and functional impairments. A first consideration is spontaneous recovery. Several studies have shown a general trend toward a decrease in cognitive difficulties over time,^81,82^ with highly variable progression profiles.^100^ Such an explanation seems unlikely here, given the absence of the effect of time elapsed since infection on baseline results. Nevertheless, we cannot distinguish between intervention-specific benefits and non-specific effects such as expectancy or placebo responses, regression to the mean, practice effects on performance tasks or other global clinical effects (e.g. social recognition of the condition, increased support and validation of symptoms). Therefore, future studies incorporating waitlist or control conditions will be essential to disentangle these effects and better determine the specific contribution of cognitive and affective tools interventions in Long COVID rehabilitation.

In addition, the sample characteristics may limit generalizability. Most participants had mild to moderate infections; only 13% were hospitalized, and the majority were females, highly educated and living in Belgium. These characteristics may reduce the applicability of the study findings, particularly to more severely infected patients in the acute phase. Furthermore, as part of the inclusion criteria, our findings are limited to Long COVID patients who reported cognitive complaints and who had sufficiently good physical condition to attend the appointments, which may also limit generalizability to all individuals affected by the disease.

Methodological choices should also be considered. For example, neuropsychological tests (e.g. BVMT-R and RBANS word-list for episodic memory) were selected for their alternative versions. They are, however, subject to a ceiling effect and are not free from practice effects, although these are smaller.^107^

Another consideration is the validity of cognitive performance and self-reported cognitive problems. Our findings are unlikely to reflect insufficient effort or disengagement during assessments. Indeed, we used embedded validity indices from the Stroop and verbal fluency tasks^108,109^ and identified eight participants with a single questionable score, but only two participants with both indices (the criterion generally recommended for identifying invalid results with high specificity^110,111^). Furthermore, validity scales of the BRIEF-A questionnaire were verified at T0 scores, and no validity score was above the threshold when calculating the mean between the two administrations of the questionnaire. However, evaluating symptom or performance validity was not the aim of this study, and our approach was not specifically designed for that purpose. Addressing this question would require a more comprehensive assessment using specific and sensitive performance validity tests.

Although infection dates were available for all participants, we did not collect information on vaccination status nor on the specific SARS-CoV-2 variant involved. Therefore, potential variant-related effects cannot be examined. However, prior studies suggest that Long COVID symptoms, and particularly cognitive impairment, occur at similar frequencies regardless of the infecting variant.^112,113^

Even though the dropout rate was low (12% at 2 months and 9% at 8 months post-intervention) and not related to the severity of complaints (i.e. dropout participants were neither the most nor the least impaired), the difference in sample sizes at the three evaluation times (for the PP analyses, *n* = 122 at baseline, *n* = 108 at 2 months and *n* = 96 at 8 months) should be considered.

While randomization is considered a gold standard for evaluating different treatments,^114^ RCTs have some limitations in psychological research, particularly in explaining why and how therapies work.^115^ Randomization can lead to sample distortions, with patient preferences significantly impacting their involvement in the therapy, which limits the generalizability of the study results.^116^ However, although our participants showed a preference for the cognitive intervention prior to randomization, no difference was observed between groups in satisfaction level, rates of discontinuation of the intervention (6 cognitive, 4 affective) or global dropout rates (15 cognitive, 13 affective).

From a qualitative perspective, patients mentioned concerns about the intervention’s structure, including the limited number of sessions (four), session duration (90 min), frequency (usually once a week) and absence of group discussions. Some patients found sessions too long due to concentration and fatigue complaints and would have preferred more time between sessions to better implement the strategies in their daily lives. These aspects should be considered for future implementation of the interventions. A qualitative study (QUA-COVCOG) is underway to further explore patients’ and clinicians’ experiences, offering deeper insight into these findings.

## Conclusions

Our study is among the first to evaluate the impact of psychoeducative interventions on cognitive and somatic difficulties in Long COVID patients, and the first to directly compare cognitive versus affective approaches. Both interventions were well-received by Long COVID patients, who considered them beneficial, though no significant interaction effects were found for most outcomes. Modest time effects were observed 2 months post-intervention for measures of cognitive complaints, objective cognitive performances (attention, working memory and long-term memory), fatigue, sleep difficulties and quality of life. Positive effects were maintained at the long-term follow-up (8 months).

Although this RCT provides promising results, further research is needed to better understand post-COVID-19 recovery trajectories, identify predictors of positive outcomes and refine clinical management strategies. Future interventions should be tailored to individual patient profiles, addressing the cognitive or affective sphere or both.

## Supplementary Material

fcaf447_Supplementary_Data

## Data Availability

Data are available on request to the authors. R script used for data analysis is available on OSF at: https://osf.io/rq53c/overview?view_only=209ea73eb1c74289b9cccfe09b5e8476.

## Appendix

### COVCOG Group members

Michel Moutschen; Gilles Dupuis; Gaël Delrue; Valentine Demoulin; Julien Goin; Clara Della Libera.

## References

[1] Salamanna F, Veronesi F, Martini L, Landini MP, Fini M. Post-COVID-19 syndrome: The persistent symptoms at the post-viral stage of the disease. A systematic review of the current data. Front Med (Lausanne). 2021;8:653516. 34017846 10.3389/fmed.2021.653516 PMC8129035

[2] Post COVID-19 condition (long COVID) . Accessed 4 September 2025. https://www.who.int/news-room/fact-sheets/detail/post-covid-19-condition-(long-covid)

[3] Peluso MJ, Deeks SG. Mechanisms of long COVID and the path toward therapeutics. Cell. 2024;187(20):5500–5529. 39326415 10.1016/j.cell.2024.07.054 PMC11455603

[4] Davis HE, McCorkell L, Vogel JM, Topol EJ. Long COVID: Major findings, mechanisms and recommendations. Nat Rev Microbiol. 2023;21(3):133–146. 36639608 10.1038/s41579-022-00846-2 PMC9839201

[5] Miskowiak KW, Pedersen JK, Gunnarsson DV, et al Cognitive impairments among patients in a long-COVID clinic: Prevalence, pattern and relation to illness severity, work function and quality of life. J Affect Disord. 2023;324:162–169. 36586593 10.1016/j.jad.2022.12.122 PMC9795797

[6] Liu TC, Yoo SM, Sim MS, Motwani Y, Viswanathan N, Wenger NS. Perceived cognitive deficits in patients with symptomatic SARS-CoV-2 and their association with post-COVID-19 condition. JAMA Netw Open. 2023;6(5):e2311974. 37145596 10.1001/jamanetworkopen.2023.11974 PMC10715897

[7] Kirchberger I, Peilstöcker D, Warm TD, et al Subjective and objective cognitive impairments in non-hospitalized persons 9 months after SARS-CoV-2 infection. Viruses. 2023;15(1):256. 36680296 10.3390/v15010256 PMC9865483

[8] Wahlgren C, Forsberg G, Divanoglou A, et al Two-year follow-up of patients with post-COVID-19 condition in Sweden: A prospective cohort study. Lancet Reg Health Eur. 2023;28:100595. 36855599 10.1016/j.lanepe.2023.100595 PMC9951394

[9] Fernandez-de-las-Peñas C, Notarte KI, Macasaet R, et al Persistence of post-COVID symptoms in the general population two years after SARS-CoV-2 infection: A systematic review and meta-analysis. J Infect. 2024;88(2):77–88. 38101521 10.1016/j.jinf.2023.12.004

[10] Carmona-Cervelló M, León-Gómez BB, Dacosta-Aguayo R, et al Long COVID: Cognitive, balance, and retina manifestations. Front Med (Lausanne). 2024;11:1–12. 10.3389/fmed.2024.1399145 PMC11260168 39036098

[11] Cabello Fernandez C, Didone V, Slama H, et al Profiles of individuals with long COVID reporting persistent cognitive complaints. Arch Clin Neuropsychol. 2025;00:1–18. doi: 10.1093/arclin/acaf064 PMC12644064 40645608

[12] Tavares-Júnior JWL, de Souza ACC, Borges JWP, et al COVID-19 associated cognitive impairment: A systematic review. Cortex. 2022;152:77–97. 35537236 10.1016/j.cortex.2022.04.006 PMC9014565

[13] Bertuccelli M, Ciringione L, Rubega M, Bisiacchi P, Masiero S, Del Felice A. Cognitive impairment in people with previous COVID-19 infection: A scoping review. Cortex. 2022;154:212–230. 35780756 10.1016/j.cortex.2022.06.002 PMC9187867

[14] Evans RA, McAuley H, Harrison EM, et al Physical, cognitive, and mental health impacts of COVID-19 after hospitalisation (PHOSP-COVID): A UK multicentre, prospective cohort study. Lancet Respir Med. 2021;9(11):1275–1287. 34627560 10.1016/S2213-2600(21)00383-0 PMC8497028

[15] Song X, Song W, Cui L, et al A comprehensive review of the global epidemiology, clinical management, socio-economic impacts, and national responses to long COVID with future research directions. Diagnostics. 2024;14(11):1–17. 10.3390/diagnostics14111168 PMC11171614 38893693

[16] Kohn L, Dauvrin M, Detollenaere J, et al Long COVID and return to work: A qualitative study. Occup Med (Chic Ill). 2024;74(1):29–36. 10.1093/occmed/kqac119 36480262

[17] Mathern R, Senthil P, Vu N, Thiyagarajan T. Neurocognitive rehabilitation in COVID-19 patients: A clinical review. South Med J. 2022;115(3):227–231. 35237843 10.14423/SMJ.0000000000001371 PMC8865030

[18] Cicerone KD, Goldin Y, Ganci K, et al Evidence-based cognitive rehabilitation: Systematic review of the literature from 2009 through 2014. Arch Phys Med Rehabil. 2019;100(8):1515–1533. 30926291 10.1016/j.apmr.2019.02.011

[19] Jeffay E, Ponsford J, Harnett A, et al INCOG 2.0 guidelines for cognitive rehabilitation following traumatic brain injury, part III: Executive functions. J Head Trauma Rehabil. 2023;38(1):52–64. 36594859 10.1097/HTR.0000000000000834

[20] Ponsford J, Velikonja D, Janzen S, et al INCOG 2.0 guidelines for cognitive rehabilitation following traumatic brain injury, part II: Attention and information processing speed. J Head Trauma Rehabil. 2023;38(1):38–51. 36594858 10.1097/HTR.0000000000000839

[21] Velikonja D, Ponsford J, Janzen S, et al INCOG 2.0 guidelines for cognitive rehabilitation following traumatic brain injury, part V: Memory. J Head Trauma Rehabil. 2023;38(1):83–102. 36594861 10.1097/HTR.0000000000000837

[22] Fine JS, Ambrose AF, Didehbani N, et al Multi-disciplinary collaborative consensus guidance statement on the assessment and treatment of cognitive symptoms in patients with post-acute sequelae of SARS-CoV-2 infection (PASC). PM R. 2022;14(1):96–111. 34902226 10.1002/pmrj.12745

[23] Möller M, Borg K, Janson C, Lerm M, Normark J, Niward K. Cognitive dysfunction in post-COVID-19 condition: Mechanisms, management, and rehabilitation. J Intern Med. 2023;294(5):563–581. 37766515 10.1111/joim.13720

[24] Heslot C, Azouvi P, Perdrieau V, Granger A, Lefèvre-Dognin C, Cogné M. A systematic review of treatments of post-concussion symptoms. J Clin Med. 2022;11(20):6224. 36294545 10.3390/jcm11206224 PMC9604759

[25] De Boussard CN, Holm LW, Cancelliere C, et al Nonsurgical interventions after mild traumatic brain injury: A systematic review. Results of the international collaboration on mild traumatic brain injury prognosis. Arch Phys Med Rehabil. 2014;(3 SUPPL):S257–S264. 24581911 10.1016/j.apmr.2013.10.009

[26] Braga LW, Oliveira SB, Moreira AS, et al Long COVID neuropsychological follow-up: Is cognitive rehabilitation relevant? NeuroRehabilitation. 2023;53(4):517–534. 38143394 10.3233/NRE-230212

[27] Hasting AS, Herzig S, Obrig H, Schroeter ML, Villringer A, Thöne-Otto AIT. The Leipzig treatment program for interdisciplinary diagnosis and therapy of neurocognitive post-COVID symptoms experiences and preliminary results. Zeitschrift fur Neuropsychologie. 2023;34(2):71–83.

[28] Assunção PF, Takahasi EHM, Amorim JAM, de Reis RL. Rehabilitation interventions in long COVID: A case report. Dement Neuropsychol. 2024;18:e20230105.

[29] Dutra LS, Shigaeff N. Proposed protocol for post COVID-19 cognitive rehabilitation for attention and memory. Dement Neuropsychol. 2024;18:e20230109. 38831970 10.1590/1980-5764-DN-2023-0109 PMC11145952

[30] Herrera E, Blanco C, Álvarez-Mundiñano B, González-Nosti M. Neuropsychological rehabilitation improves memory and action naming in patients with post-COVID-19 syndrome. Am J Speech Lang Pathol. 2024;33(2):791–799. 38118457 10.1044/2023_AJSLP-23-00157

[31] García-Molina A, García-Carmona S, Espiña-Bou M, Rodríguez-Rajo P, Sánchez-Carrión R, Enseñat-Cantallops A. Neuropsychological rehabilitation for post–COVID-19 syndrome: Results of a clinical programme and six-month follow up. Neurología (Engl Ed). 2024;39(7):592–603. 36116770 10.1016/j.nrleng.2022.06.007 PMC9476330

[32] De Groot MH, Phillips SJ, Eskes GA. Fatigue associated with stroke and other neurologic conditions: Implications for stroke rehabilitation. Arch Phys Med Rehabil. 2003;84(11):1714–1720. 14639575 10.1053/s0003-9993(03)00346-0

[33] Brode WM, Melamed E. A practical framework for Long COVID treatment in primary care. Life Sci. 2024;354:122977. 39142509 10.1016/j.lfs.2024.122977

[34] Managing fatigue in adults after acquired brain injury | 48 | Neuropsy. Accessed 19 March 2025. https://www.taylorfrancis.com/chapters/edit/10.4324/9781315629537-48/managing-fatigue-adults-acquired-brain-injury-donna-malley

[35] Ghali A, Lacombe V, Ravaiau C, et al The relevance of pacing strategies in managing symptoms of post-COVID-19 syndrome. J Transl Med. 2023;21(1):375. 37291581 10.1186/s12967-023-04229-w PMC10248991

[36] Parker M, Sawant HB, Flannery T, et al Effect of using a structured pacing protocol on post-exertional symptom exacerbation and health status in a longitudinal cohort with the post-COVID-19 syndrome. J Med Virol. 2023;95(1):e28373. 36461167 10.1002/jmv.28373 PMC9878088

[37] Kuut TA, Müller F, Csorba I, et al Efficacy of cognitive-behavioral therapy targeting severe fatigue following coronavirus disease 2019: Results of a randomized controlled trial. Clin Infect Dis. 2023;77(5):687–695. 37155736 10.1093/cid/ciad257 PMC10495128

[38] Taquet M, Geddes JR, Husain M, Luciano S, Harrison PJ. 6-month neurological and psychiatric outcomes in 236 379 survivors of COVID-19: A retrospective cohort study using electronic health records. Lancet Psychiatry. 2021;8(5):416–427. 33836148 10.1016/S2215-0366(21)00084-5 PMC8023694

[39] Huang L, Yao Q, Gu X, et al 1-year outcomes in hospital survivors with COVID-19: A longitudinal cohort study. The Lancet. 2021;398(10302):747–758. 10.1016/S0140-6736(21)01755-4 PMC8389999 34454673

[40] Seighali N, Abdollahi A, Shafiee A, et al The global prevalence of depression, anxiety, and sleep disorder among patients coping with post COVID-19 syndrome (long COVID): A systematic review and meta-analysis. BMC Psychiatry. 2024;24(1):1–13. 38321404 10.1186/s12888-023-05481-6 PMC10848453

[41] Dotson VM . Unique and interactive effect of anxiety and depressive symptoms on cognitive and brain function in young and older adults. J Depress Anxiety. 2014;S1(01):1–23. 10.4172/2167-1044.S1-003 PMC4222514 25383262

[42] Poletti S, Palladini M, Mazza MG, et al Long-term consequences of COVID-19 on cognitive functioning up to 6 months after discharge: Role of depression and impact on quality of life. Eur Arch Psychiatry Clin Neurosci. 2022;272(5):773–782. 34698871 10.1007/s00406-021-01346-9 PMC8546751

[43] Terpstra AR, Cairncross M, Yeates KO, et al Psychological mediators of avoidance and endurance behavior after concussion. Rehabil Psychol. 2021;66(4):470–478. 34410757 10.1037/rep0000390 PMC8648930

[44] Al Sayegh A, Sandford D, Carson AJ. Psychological approaches to treatment of postconcussion syndrome: A systematic review. J Neurol Neurosurg Psychiatry. 2010;81(10):1128–1134. 20802219 10.1136/jnnp.2008.170092

[45] Sullivan KA, Kaye SA, Blaine H, et al Psychological approaches for the management of persistent postconcussion symptoms after mild traumatic brain injury: A systematic review. Disabil Rehabil. 2020;42(16):2243–2251. 30741023 10.1080/09638288.2018.1558292

[46] Ponsford J, Lee NK, Wong D, et al Efficacy of motivational interviewing and cognitive behavioral therapy for anxiety and depression symptoms following traumatic brain injury. Psychol Med. 2016;46(5):1079–1090. 26708017 10.1017/S0033291715002640

[47] Gómez-De-Regil L, Estrella-Castillo DF, Vega-Cauich J. Psychological intervention in traumatic brain injury patients. Behav Neurol. 2019;2019: 1–8. 10.1155/2019/6937832 PMC6525953 31191738

[48] Huth D, Bräscher AK, Tholl S, et al Cognitive-behavioral therapy for patients with post-COVID-19 condition (CBT-PCC): A feasibility trial. Psychol Med. 2024;54(6):1122–1132. 37842765 10.1017/S0033291723002921

[49] Koller K, Kastel-Hoffmann S, Herold R, et al A prospective non-randomized controlled trial testing the effectiveness of psychotherapeutic inpatient treatment of post-COVID-19 syndrome—Study protocol. BMC Psychol. 2024;12(1):486. 39285491 10.1186/s40359-024-01974-5 PMC11404027

[50] Skilbeck L . Patient-led integrated cognitive behavioural therapy for management of long COVID with comorbid depression and anxiety in primary care—A case study. Chronic Illn. 2022;18(3):691–701. 35821571 10.1177/17423953221113605

[51] Compagno S, Palermi S, Pescatore V, et al Physical and psychological reconditioning in long COVID syndrome: Results of an out-of-hospital exercise and psychological—Based rehabilitation program. Int J Cardiol Heart Vasc. 2022;41:101080. 35854691 10.1016/j.ijcha.2022.101080 PMC9286763

[52] Ferrario SR, Panzeri A, Cerutti P, Sacco D. The psychological experience and intervention in post-acute COVID-19 inpatients. Neuropsychiatr Dis Treat. 2021;17:413–422. 33603379 10.2147/NDT.S283558 PMC7884934

[53] Hausswirth C, Schmit C, Rougier Y, Coste A. Positive impacts of a four-week neuro-meditation program on cognitive function in post-acute sequelae of COVID-19 patients: A randomized controlled trial. Int J Environ Res Public Health. 2023;20(2):1–16. 10.3390/ijerph20021361 PMC9858974 36674117

[54] Willems S, Didone V, Cabello Fernandez C, et al COVCOG: Immediate and long-term cognitive improvement after cognitive versus emotion management psychoeducation programs—A randomized trial in COVID patients with neuropsychological difficulties. BMC Neurol. 2023;23(1):307. 37596541 10.1186/s12883-023-03346-9 PMC10436391

[55] Randolph C, Tierney MC, Mohr E, Chase TN. The repeatable battery for the assessment of neuropsychological status (RBANS): Preliminary clinical validity. J Clin Exp Neuropsychol. 1998;20(3):310–319. 9845158 10.1076/jcen.20.3.310.823

[56] Benedict RHB, Groninger L, Schretlen D, Dobraski M, Shpritz B. Revision of the brief visuospatial memory test: Studies of normal performance, reliability, and validity. Psychol Assess. 1996;8(2):145–153.

[57] Zimmermann P, Fimm B. A test battery for attentional performance. Applied neuropsychology of attention. Psychology Press; 2011.

[58] Brickenkamp R, Schmidt-Atzert L, Liepmann D. D2-R: Test d’attention concentrée révisé. Accessed 20 March 2025. https://www.hogrefe.com/fr/shop/test-d-attention-concentree.html.

[59] Azouvi P, Vallat-Azouvi C, Joseph PA, et al Executive functions deficits after severe traumatic brain injury: The GREFEX study. J Head Trauma Rehabil. 2016;31(3):E10–E20. 10.1097/HTR.0000000000000169 26394296

[60] Geurten M, Vincent E, Van Der Linden M, et al Working memory assessment: Construct validity of the Brown-Peterson test. Can J Behav Sci/Revue canadienne des sciences. 2016;48(4):328–336.

[61] Nasreddine ZS, Phillips NA, Bédirian V, et al The Montreal cognitive assessment, MoCA: A brief screening tool for mild cognitive impairment. J Am Geriatr Soc. 2005;53(4):695–699. 15817019 10.1111/j.1532-5415.2005.53221.x

[62] Waid-Ebbs JK, Wen PS, Heaton SC, et al The item level psychometrics of the behaviour rating inventory of executive function-adult (BRIEF-A) in a TBI sample. Brain Inj. 2012;26(13–14):1646–1657. 22876936 10.3109/02699052.2012.700087

[63] Troyer AK, Rich JB. Psychometric properties of a new metamemory questionnaire for older adults. J Gerontol B Psychol Sci Soc Sci. 2002;57(1):19–27. 10.1093/geronb/57.1.p19 11773220

[64] Multiple Sclerosis Council for Clinical Practice Guidelines . Fatigue and multiple sclerosis: Evidence-based management strategies for fatigue in multiple sclerosis. Paralyzed Veterans of America; 1998.

[65] Buysse DJ, Reynolds CF, Monk TH, Berman SR, Kupfer DJ. The Pittsburgh sleep quality Index: A new instrument for psychiatric practice and research. Psychiatry Res. 1989;28:193–213. 2748771 10.1016/0165-1781(89)90047-4

[66] Lambert MJ, Burlingame GM, Umphress V, et al The reliability and validity of the outcome questionnaire. Clin Psychol Psychother. 1996;3(4):249–258.

[67] Duquette RL, Dupuis G, Perrault J. A new approach for quality of life assessment in cardiac patients: Rationale and validation of the quality of life systemic inventory. Can J Cardiol. 1994;10(1):106–112. 8111664

[68] EuroQol Research Foundation . EQ-5D-5L user guide, 2019. Accessed 20 March 2025. https://euroqol.org/publications/user-guides

[69] Reilly MC, Zbrozek AS, Dukes EM. The validity and reproducibility of a work productivity and activity impairment instrument. Pharmacoeconomics. 1993;4(5):353–365. 10146874 10.2165/00019053-199304050-00006

[70] Levine B, Robertson IH, Clare L, et al Rehabilitation of executive functioning: An experimental–clinical validation of goal management training. J Int Neuropsychol Soc. 2000;6(3):299–312. 10824502 10.1017/s1355617700633052

[71] Cantin JF, Ouellet MC, Turcotte N, Lessard J, Potvin I, Boutin N. Le guide de l’énergie vers une meilleure gestion de la fatigue: guide de l’intervenant, outils d’évaluation. Institut de réadaptation en déficience physique de Québec; 2014. Accessed 20 March 2025. https://www.cassetete22.com/wp-content/uploads/2017/06/Guide-de-l%C3%A9nergie-Guide-de-lintervenant-Par-l%E2%80%99-IRDPQ-Quebec.pdf

[72] Fasotti L, Kovacs F, Eling PATM, Brouwer WH. Time pressure management as a compensatory strategy training after closed head injury. Neuropsychol Rehabil. 2000;10(1):47–65.

[73] Kaplan SJ . The private practice of behavior therapy: A guide for behavioral practitioners. Springer Nature; 1986.

[74] Dugas MJ, Gagnon F, Ladouceur R, Freeston MH. Generalized anxiety disorder: A preliminary test of a conceptual model. Behav Res Ther. 1998;36(2):215–226. 9613027 10.1016/s0005-7967(97)00070-3

[75] Fanget F, Rouchouse B. Affirmation de soi (L’): Une méthode de thérapie. Odile Jacob; 2007.

[76] Blairy S, Baeyens C, Wagener A. L’activation comportementale: Traitement des évitements comportementaux et de la rumination mentale. Mardaga; 2020.

[77] Cohen J . Statistical power analysis for the behavioral sciences. Routledge; 2013.

[78] R Core Team . R: A language and environment for statistical computing. (Version 4.1); 2021. Accessed 20 March 2025. Retrieved from https://www.R-project.org/

[79] Maronna RA, Zamar RH. Robust estimates of location and dispersion for high-dimensional datasets. Technometrics. 2002;44(4):307–317.

[80] Gnanadesikan R, Kettenring JR. Robust estimates, residuals, and outlier detection with multiresponse data. Biometrics. 1972;28:81–124.

[81] Badinlou F, Abzhandadze T, Rahimian F, Jansson-Fröjmark M, Hedman-Lagerlöf M, Lundgren T. Investigating the trajectory of post-COVID impairments: A longitudinal study in Sweden. Front Psychol. 2024;15:1402750. 38915427 10.3389/fpsyg.2024.1402750 PMC11195806

[82] Diana L, Regazzoni R, Sozzi M, et al Monitoring cognitive and psychological alterations in COVID-19 patients: A longitudinal neuropsychological study. J Neurol Sci. 2023;444:120511. 36473347 10.1016/j.jns.2022.120511 PMC9707027

[83] Potter SDS, Brown RG, Fleminger S. Randomised, waiting list controlled trial of cognitive-behavioural therapy for persistent postconcussional symptoms after predominantly mild-moderate traumatic brain injury. J Neurol Neurosurg Psychiatry. 2016;87(10):1075–1083. 27496149 10.1136/jnnp-2015-312838

[84] Uiterwijk D, Stargatt R, Humphrey S, Crowe SF. The relationship between cognitive functioning and symptoms of depression, anxiety, and post-traumatic stress disorder in adults with a traumatic brain injury: A meta-analysis. Neuropsychol Rev. 2022;32(4):758–806. 34694543 10.1007/s11065-021-09524-1

[85] Gulick SH, Mandel S, Maitz EA, Brigham CR, Direnfeld LK. Cognitive screening tools. AMA Guides Newsletter. 2021;26(3):3–7.

[86] Navas-Otero A, Calvache-Mateo A, Calles-Plata I, et al A lifestyle adjustments program in long COVID-19 improves symptomatic severity and quality of life. A randomized control trial. Patient Educ Couns. 2024;122:108180. 38330704 10.1016/j.pec.2024.108180

[87] Ito D, Watanabe A, Takeichi S, Ishihara A, Yamamoto K. A preliminary study of work-focused cognitive behavioural group therapy for Japanese workers. Behav Cogn Psychother. 2019;47(2):251–256. 29871705 10.1017/S1352465818000280

[88] Liu EN, Yang JH, Patel L, et al Longitudinal analysis and treatment of neuropsychiatric symptoms in post-acute sequelae of COVID-19. J Neurol. 2023;270(10):4661–4672. 37493802 10.1007/s00415-023-11885-x PMC10910663

[89] Rass V, Beer R, Schiefecker AJ, et al Neurological outcomes 1 year after COVID-19 diagnosis: A prospective longitudinal cohort study. Eur J Neurol. 2022;29(6):1685–1696. 35239247 10.1111/ene.15307 PMC9111823

[90] Davis HE, Assaf GS, McCorkell L, et al Characterizing long COVID in an international cohort: 7 months of symptoms and their impact. EClinicalMedicine. 2021;38:101019. 34308300 10.1016/j.eclinm.2021.101019 PMC8280690

[91] Deng J, Zhou F, Hou W, et al The prevalence of depression, anxiety, and sleep disturbances in COVID-19 patients: A meta-analysis. Ann N Y Acad Sci. 2021;1486(1):90–111. 33009668 10.1111/nyas.14506 PMC7675607

[92] Kerksieck P, Ballouz T, Haile SR, et al Post COVID-19 condition, work ability and occupational changes in a population-based cohort. Lancet Reg Health—Eur. 2023;31:100671. 37366496 10.1016/j.lanepe.2023.100671 PMC10287546

[93] Audrit H, Beauchamp M, Tinawi S, et al Multidimensional psychoeducative and counseling intervention (SAAM) for symptomatic patients with mild traumatic brain injury: A pilot randomized controlled trial. J Head Trauma Rehabil. 2021;36(4):E249–E261. 33656475 10.1097/HTR.0000000000000653

[94] Azariah A, Watanabe T. Postconcussion syndrome assessment, management, and treatment. Elsevier Inc.; 2020.

[95] Garrido Ardila EM, Rodríguez Mansilla J, Jiménez Palomares M, Torres Piles ST, González López-Arza MV. Cognitive rehabilitation in fibromyalgia and chronic fatigue syndrome: Current advances. In: Columbus AM, ed. Advances in psychology research. Nova Science Publishers; 2017:1–14.

[96] Sacks-Zimmerman A, Bergquist TF, Farr EM, Cornwell MA, Kanellopoulos D. Rehabilitation of neuropsychiatric symptoms in patients with Long COVID: Position statement. Arch Phys Med Rehabil. 2023;104(2):350–354. 36272444 10.1016/j.apmr.2022.10.001 PMC9581644

[97] Espinoza C, Martella D. Cognitive functions in COVID-19 survivors, approaches strategies, and impact on health systems: A qualitative systematic review. Springer Berlin Heidelberg; 2023. 10.1007/s00406-023-01662-2 37648954

[98] Floridia M, Giuliano M, Weimer LE, et al Symptom profile, case and symptom clustering, clinical and demographic characteristics of a multicentre cohort of 1297 patients evaluated for long-COVID. BMC Med. 2024;22(1):532. 39543596 10.1186/s12916-024-03746-9 PMC11566432

[99] Voruz P, Cionca A, De Alcântara I J, et al Functional connectivity underlying cognitive and psychiatric symptoms in post-COVID-19 syndrome: Is anosognosia a key determinant? Brain Commun. 2022;4(2):1–15. 10.1093/braincomms/fcac057 PMC8956133 35350554

[100] Voruz P, de Alcântara IJ, Nuber-Champier A, et al Persistence and emergence of new neuropsychological deficits following SARS-CoV-2 infection: A follow-up assessment of the Geneva COVID-COG cohort. J Glob Health. 2024;14: 05008. 38452292 10.7189/jogh.14.05008 PMC10919907

[101] Gómez Bravo R, Infanti A, Billieux J, et al Unmasking the psychological landscape of Long COVID: A cluster-analytical approach. Biopsychosoc Sci Med. 2025;87(3):162–172. 39964227 10.1097/PSY.0000000000001380 PMC11957433

[102] Amsterdam D, Kupershmidt A, Avinir A, et al Long COVID-19 Enigma: Unmasking the role of distinctive personality profiles as risk factors. J Clin Med. 2024;13(10):2886. 38792428 10.3390/jcm13102886 PMC11122355

[103] Gloeckl R, Leitl D, Schneeberger T, Jarosch I, Koczulla AR. Rehabilitative interventions in patients with persistent post COVID-19 symptoms—A review of recent advances and future perspectives. Eur Arch Psychiatry Clin Neurosci. 2023;274(8):1819–1828. 37326700 10.1007/s00406-023-01631-9 PMC11579067

[104] Duff EP, Zetterberg H, Heslegrave A, et al Plasma proteomic evidence for increased Alzheimer’s disease-related brain pathology after SARS-CoV-2 infection. Nat Med. 2024;31:797-806. 10.1038/s41591-024-03426-4 PMC11922756 39885359

[105] Levine KS, Leonard HL, Blauwendraat C, et al Virus exposure and neurodegenerative disease risk across national biobanks. Neuron. 2023;111(7):1086–1093.e2. 36669485 10.1016/j.neuron.2022.12.029 PMC10079561

[106] Bird M, Ouellette C, Whitmore C, et al Preparing for patient partnership: A scoping review of patient partner engagement and evaluation in research. Health Expect. 2020;23(3):523–539. 32157777 10.1111/hex.13040 PMC7321722

[107] Beglinger LJ, Gaydos B, Tangphao-Daniels O, et al Practice effects and the use of alternate forms in serial neuropsychological testing. Arch Clin Neuropsychol. 2005;20(4):517–529. 15896564 10.1016/j.acn.2004.12.003

[108] Arentsen TJ, Boone KB, Lo TTY, et al Effectiveness of the Comalli Stroop test as a measure of negative response bias. Clin Neuropsych. 2013;27(6):1060–1076. 10.1080/13854046.2013.803603 23742292

[109] Sugarman MA, Axelrod BN. Embedded measures of performance validity using verbal fluency tests in a clinical sample. Appl Neuropsychol. 2015;22(2):141–146. 10.1080/23279095.2013.873439 25153155

[110] Chafetz M . Reducing the probability of false positives in malingering detection of social security disability claimants detection of social security disability claimants. Clin Neuropsychol. 2011;25:1239–1252. 21722051 10.1080/13854046.2011.586785

[111] Bertrand JA, Chartrand JP, Gauthier AK, Kennepohl S. Validité de performance aux tests neuropsychologiques : Prise de position de l ‘association québécoise des neuropsychologues. Neuropsychologie clinique et appliquée 2023;5: 1–16.().

[112] de Erausquin GA, Zwir JI, Snyder HM, et al Cognitive sequealae of COVID-19 is not predicted by SARS-CoV-2 variants. Alzheimer’s Dement. 2023;19(S24):1–2.

[113] Saigal A, Nagoda Niklewicz C, Naidu SB, et al Cross-sectional study evaluating the impact of SARS-CoV-2 variants on Long COVID outcomes in UK hospital survivors. BMJ Open Respir Res. 2023;10(1):1–12. 10.1136/bmjresp-2023-001667 PMC10401240 37536948

[114] Charlton BG . Medical practice and the double-blind, randomized controlled trial. Br J Gen Pract. 1991;41(350):355–356. 1793642 PMC1371714

[115] Carey TA, Stiles WB. Some problems with randomized controlled trials and some viable alternatives. Clin Psychol Psychother. 2016;23(1):87–95. 25601435 10.1002/cpp.1942

[116] Brewin C, Bradley C. Patient preferences and randomised controlled trials. Bmj. 1989;299:313–315. 2504416 10.1136/bmj.299.6694.313 PMC1837157

