# Supplementary material for: Cognitive and affective psychoeducation for Long COVID: a randomized controlled trial
Source: Brain Commun. 2025 Nov 11;7(6):fcaf447. doi: 10.1093/braincomms/fcaf447 (PMC12631555; doi:10.1093/braincomms/fcaf447)
Supplement: fcaf447_Supplementary_Data [file fcaf447_supplementary_data.docx]

**SUPPLEMENTARY MATERIAL**

**Supplementary information 1. Details on timing of follow-up deviation from protocol**

For the short-term follow-up, 55 patients had irregular intervals between the end of the intervention and their evaluation: 16 patients were assessed slightly early (<52 days; mean: 47; range: 38–51), and 39 too late (>69 days; mean: 91; range: 70–178). Similarly, at the long-term follow-up, 6 patients were evaluated too early (<228 days; mean: 223; range: 216–227), and 21 patients too late (>259 days; mean: 270; range: 260–297).

**Supplementary table 1 Descriptive sociodemographic characteristics, COVID-19 history and premorbidities of 122 patients following ITT principles**

|  |  | Total (*N*=122) | Cognitive intervention (*N*=63) | Affective intervention (*N*=59) |
| --- | --- | --- | --- | --- |
| ***Demographic factors*** | Age (mean ± *SD*) [range] | 47 ± 10 [21-66] | 47.3 ± 9.8 [22-65] | 46.7 ± 10.5 [21-66] |
|  | Sex (female) | 85 (69.7%) | 43 (68.3%) | 42 (71.2%) |
|  | Years of education (mean ± *SD*) [range] | 14 ± 3 [6-17] | 14.2 ± 3 [6-17] | 13.9 ± 3 [6-17] |
|  | Actively employed/student before infection | 114 (93.4%) | 60 (95.2%) | 54 (91.5%) |
|  | Actively employed/student at study inclusion | 73 (59.8%) | 34 (54%) | 39 (66.1%) |
|  | On sick leave before infection | 2 (1.6%) | 1 (1.6%) | 1 (1.7%) |
|  | On sick leave at study inclusion | 41 (33.6%) | 24 (38.1%) | 17 (28.8%) |
| ***COVID-19 history^1^*** | Asymptomatic | 1 (0.8%) | 0 | 1 (1.7%) |
|  | Mild infection | 67 (54.9%) | 35 (55.6%) | 32 (54.2%) |
|  | Moderate infection | 41 (33.6%) | 21 (33.3%) | 20 (33.9%) |
|  | Severe infection | 13 (10.7%) | 7 (11.1%) | 6 (10.2%) |
|  | Hospitalized | 16 (13.1% ; 10 female) | 9 (14.3% ; 6 female) | 7 (11.9% ; 4 female) |
|  | ICU treatment; mean stay | 8 (6.6% ; 3 female) ; 13 days | 5 (7.9% ; 2 female) ; 16 days | 3 (5.1% ; 1 female) ; 10 days |
|  | Number of infections (mean ± *SD*) [range] | 1.7 ± 0.9 [1–5] | 1.7 ± 0.9 [1–4] | 1.7 ± 0.9 [1–5] |
|  | Time since first infection (months) | 20.9 ± 8.6 [4–39] | 22.6 ± 8.4 [4–39] | 19.1 ± 8.6 [4–34] |
| ***Premorbidities^2^*** | Endocrine, nutritional, or metabolic diseases | 34% | 37% | 32% |
|  | Mental and Behavioural Disorders | 19% | 16% | 22% |
|  | Diseases of the circulatory system | 17% | 16% | 19% |
|  | Surgical operations | 11% | 10% | 14% |
|  | Sleep-wake cycle disorders | 10% | 13% | 7% |
|  | Nervous system diseases | 8% | 10% | 7% |
|  | Respiratory system diseases | 8% | 5% | 12% |
|  | Neurodevelopmental disorders | 6% | 6% | 5% |
|  | Infectious or parasitic diseases | 6% | 2% | 10% |
|  | Chronic pain | 5% | 2% | 8% |
|  | Diseases of the ear or mastoid process | 5% | 3% | 7% |

**Notes**: ^1^ According to the criteria of the National Institutes of Health guideline for acute infection severity: Asymptomatic or presymptomatic infection: Individuals who test positive for SARS-CoV-2 using a virologic test (i.e. a nucleic acid amplification test [NAAT] or an antigen test) but have no symptoms consistent with COVID-19; Mild illness: Individuals who have any of the various signs and symptoms of COVID-19 (e.g. fever, cough, sore throat, malaise, headache, muscle pain, nausea, vomiting, diarrhoea, loss of taste and smell) but do not have shortness of breath, dyspnoea, or abnormal chest imaging; Moderate illness: Individuals who show evidence of lower respiratory disease during clinical assessment or imaging and who have an oxygen saturation measured by pulse oximetry (SpO_2_) ≥94% on room air at sea level; Severe illness: Individuals who have an SpO_2_ <94% on room air at sea level, a ratio of arterial partial pressure of oxygen to fraction of inspired oxygen (PaO_2_/FiO_2_) <300 mm Hg, a respiratory rate >30 breaths/min, or lung infiltrates >50%.

^2^ Premorbidities were based on the International Classification of Diseases (ICD-11; WHO, 2019); total premorbidities with a frequency <5% are not displayed. ICU: Intensive care unit.

**Supplementary table 2. Descriptive sociodemographic characteristics, COVID-19 history and premorbidities of 108 patients based on PP approach**

|  |  | Total |
| --- | --- | --- |
| ***Demographic factors*** | Age (mean ± *SD*) [range] | 47 ± 10 [21-65] |
|  | Sex (female) | 75 (69.4%) |
|  | Years of education (mean ± *SD*) [range] | 14 ± 3 [6-17] |
|  | Actively employed/student before infection | 101 (93.5%) |
|  | Actively employed/student at study inclusion | 63 (58.3%) |
|  | On sick leave before infection | 2 (1.9%) |
|  | On sick leave at study inclusion | 39 (36.1%) |
| ***COVID-19^1^*** | Asymptomatic | 0 |
|  | Mild infection | 57 (52.8%) |
|  | Moderate infection | 39 (36.1%) |
|  | Severe infection | 12 (11.1%) |
|  | Hospitalized | 15 (13.9% ; 9 female) |
|  | ICU treatment; mean stay | 8 (7.4% ; 3 female) ; 13 days |
|  | Number of infections (mean ± *SD*) [range] | 1.7 ± 0.9 [1–5] |
|  | Time since first infection (months) | 20.6 ± 8.4 [4–39] |
| ***Premorbidities^2^*** | Endocrine, nutritional or metabolic diseases | 35% |
|  | Mental and Behavioural Disorders | 21% |
|  | Diseases of the circulatory system | 17% |
|  | Surgical operations | 14% |
|  | Sleep-wake cycle disorders | 11% |
|  | Nervous system diseases | 10% |
|  | Neurodevelopmental disorders | 10% |
|  | Respiratory system diseases | 8% |
|  | Infectious or parasitic diseases | 7% |
|  | Chronic pain | 6% |
|  | Diseases of the ear or mastoid process | 6% |

**Notes**: ^1^ According to the criteria of the National Institutes of Health guideline for acute infection severity: Asymptomatic or presymptomatic infection: Individuals who test positive for SARS-CoV-2 using a virologic test (i.e. a nucleic acid amplification test [NAAT] or an antigen test) but have no symptoms consistent with COVID-19; Mild illness: Individuals who have any of the various signs and symptoms of COVID-19 (e.g. fever, cough, sore throat, malaise, headache, muscle pain, nausea, vomiting, diarrhoea, loss of taste and smell) but do not have shortness of breath, dyspnoea, or abnormal chest imaging; Moderate illness: Individuals who show evidence of lower respiratory disease during clinical assessment or imaging and who have an oxygen saturation measured by pulse oximetry (SpO_2_) ≥94% on room air at sea level; Severe illness: Individuals who have an SpO_2_ <94% on room air at sea level, a ratio of arterial partial pressure of oxygen to fraction of inspired oxygen (PaO_2_/FiO_2_) <300 mm Hg, a respiratory rate >30 breaths/min, or lung infiltrates >50%.

^2^ Premorbidities were based on the International Classification of Diseases (ICD-11; WHO, 2019); total premorbidities with a frequency <5% are not displayed. ICU: Intensive care unit.

**Supplementary table 3. Results at 2 months follow-up for LMM on cognitive complaints following a PP approach**

|  | Time effect | | AFFECTIVE  INTERVENTION | | | | COGNITIVE  INTERVENTION | | | | Time by group interaction | | | |
| --- | --- | --- | --- | --- | --- | --- | --- | --- | --- | --- | --- | --- | --- | --- |
|  | *F* | *p* | Baseline (Mean ± *SD*) | 2 months post-intervention (Mean ± *SD*) | *d* (Time effect) | CI95 | Baseline (Mean ± *SD*) | 2 months post-intervention (Mean ± *SD*) | *d* (Time effect) | CI95 | *d* (interaction effect) | CI95 | *F* | *p* |
| ***BRIEF (GEC)*** | 16.9 | <0.001 | 137.5 (± 22.2) | 132.1 (± 26.9) | 0.51 | [0.15, 0.88] | 140.2 (± 20.6) | 134.9 (± 21) | 0.54 | [0.18, 0.9] | 0.002 | [-0.05, 0.05] | 0.01 | 0.92 |
| ***MMQ (composite score)*** | 16.3 | <0.001 | 94.3 (± 20.6) | 100.9 (± 23) | 0.54 | [0.17, 0.91] | 94.7 (± 16.9) | 104.4 (± 19.1) | 0.49 | [0.14, 0.85] | 0.006 | [-0.06, 0.07] | 0.03 | 0.85 |

Notes. GEC= Global Executive Composite; BRI= Behavioural Regulation Index; MI= Metacognition Index.

**Supplementary table 4. Results at 2-month follow-up for LMM on cognitive and somatic complaints, following a PP approach**

|  | Time effect | | AFFECTIVE  INTERVENTION | | | | COGNITIVE  INTERVENTION | | | | Time-by-group interaction | | | |
| --- | --- | --- | --- | --- | --- | --- | --- | --- | --- | --- | --- | --- | --- | --- |
|  | *F* | *p* | Baseline (Mean ± *SD*) | 2 months post-intervention (Mean ± *SD*) | *d* (Time effect) | CI95 | Baseline (Mean ± *SD*) | 2 months post-intervention (Mean ± *SD*) | *d* (Time effect) | CI95 | *d* (interaction effect) | CI95 | *F* | *p* |
| ***BRIEF BRI*** | 11.1 | 0.001 | 54.9 (± 11.2) | 53.1 (± 12.6) | 0.45 | [0.08, 0.81] | 57.3 (± 10.4) | 55.5 (± 10.4) | 0.41 | [0.05, 0.76] | 0.001 | [-0.08, 0.1] | 0.02 | 0.89 |
| ***BRIEF MI*** | 15.8 | <0.001 | 82.6 (± 13.1) | 79 (±16) | 0.59 | [0.23, 0.96] | 82.9 (± 12.9) | 79.4 (± 12.6) | 0.43 | [0.07, 0.78] | 0.04 | [-0.08, 0.14] | 0.43 | 0.51 |
| ***MMQ Satisfaction*** | 14.4 | <0.001 | 21.8 (±12.7) | 24.8 (± 13.5) | 0.44 | [0.07, 0.81] | 19.1 (± 10.2) | 22.7 (± 11.1) | 0.53 | [0.18, 0.89] | -0.02 | [-0.16, 0.12] | 0.1 | 0.72 |
| ***MMQ Ability*** | 20.1 | <0.001 | 32.9 (± 12.1) | 36.3 (±13.2) | 0.51 | [0.14, 0.88] | 33.8 (±10.8) | 37.6 (±11.7) | 0.64 | [0.28, 0.99] | -0.04 | [-0.16, 0.1] | 0.3 | 0.61 |
| ***MMQ Strategies*** | 5.7 | 0.02 | 39.6 (± 12.6) | 39.8 (± 11.5) | 0.03 | [-0.33, 0.4] | 41.6 (± 10.5) | 44.2 (± 9.7) | 0.36 | [0.004, 0.71] | -0.06 | [-0.16, 0.04] | 1.6 | 0.21 |
| ***QLSI (total score)*** | 12.9 | <0.001 | 10.3 (±7.7) | 7.5 (±7.3) | 0.38 | [-0.75, -0.02] | 12.8 (± 8.5) | 8.9 (±7.5) | 0.54 | [-0.89, -0.18] | 0.06 | [-0.12, 0.22] | 0.3 | 0.56 |
| ***MFIS (physical fatigue)*** | 13.3 | <0.001 | 26.4 (±7.1) | 23.5 (±9) | 0.55 | [-0.92, -0.18] | 27.2 (±6.8) | 24.7 (±8) | 0.38 | [-0.74, -0.03] | -0.06 | [-0.22, 0.1] | 0.4 | 0.51 |
| ***MFIS (cognitive fatigue)*** | 20.6 | <0.001 | 30.4 (±6.4) | 26.6 (±9.7) | 0.64 | [-1, -0.27] | 30.6 (±6.2) | 26.9 (±8.3) | 0.52 | [-0.88, -0.17] | -0.04 | [-0.24, 0.14] | 0.2 | 0.64 |
| ***MFIS (psychosocial fatigue)*** | 2.8 | 0.1 | 5.2 (±2.5) | 5.1 (±2.2) | 0.05 | [-0.32, 0.41] | 5.7 (±1.9) | 4.8 (±2.3) | 0.48 | [-0.84, -0.12] | 0.2 | [0, 0.41] | 4.2 | 0.04 |
| ***MFIS (total score)*** | 20.0 | <0.001 | 61.9 (± 13.8) | 55.3 (± 19.2) | 0.6 | [-0.97, -0.23] | 63.5 (±12.6) | 56.4 (± 17) | 0.54 | [-0.9, -0.19] | -0.02 | [-0.2, 0.16] | 0.1 | 0.82 |
| ***PSQI (total score)*** | 5.4 | 0.02 | 8.7 (± 3.9) | 8.1 (±3.5) | 0.22 | [-0.59, 0.14] | 9.7 (±4) | 8.6 (±4) | 0.38 | [-0.73, -0.02] | 0.04 | [-0.12, 0.22] | 0.4 | 0.55 |
| ***OQ-45 (symptom distress)*** | 7.2 | 0.008 | 42.8 (± 15.7) | 39 (± 15.8) | 0.18 | [-0.54, 0.19] | 46.5 (± 15.8) | 42.7 (±15.8) | 0.51 | [-0.87, -0.16] | 0.1 | [-0.04, 0.24] | 1.7 | 0.19 |
| ***OQ-45 (interpersonal relations)*** | 0.2 | 0.68 | 11.3 (±5.8) | 11.8 (±6.3) | -0.46 | [0.09, 0.82] | 12.5 (±6.9) | 11.8 (±6.2) | 0.35 | [-0.71,0] | 0.26 | [0.1,0.43] | 10 | 0.002 |
| ***OQ-45 (social role)*** | 0.8 | 0.36 | 13.3 (±4.7) | 12.4 (±5.3) | 0.02 | [-0.38,0.35] | 13.5 (±4.9) | 12.8 (±5.4) | 0.22 | [-0.57,0.14] | 0.08 | [-0.12,0.26] | 0.6 | 0.44 |
| ***OQ-45 (total score)*** | 3.5 | 0.06 | 67.4 (±22.4) | 63.2 (±24.4) | -0.008 | [-0.36,0.37] | 72.5 (±23.7) | 67.3 (±23.7) | 0.49 | [-0.84,-0.13] | 0.16 | [0, 0.3] | 3.8 | 0.05 |
| ***WPAI (work impairment)*** | 7.6 | 0.007 | 57% (±32) | 45% (±28) | 0.57 | [-1.02, -0.13] | 51% (±32) | 48% (±28) | 0.33 | [-0.8, 0.15] | -0.08 | [-0.26, 0.12] | 0.6 | 0.45 |
| ***WPAI (activity impairment)*** | 3.3 | 0.07 | 55.4% (±27.4) | 54% (±30) | 0.15 | [-0.51, 0.22] | 60% (±27) | 54% (±27) | 0.32 | [-0.67, 0.04] | 0.06 | [-0.1, 0.2] | 0.5 | 0.5 |
| ***EQ-5D-5L (VAS score)*** | 10.3 | 0.002 | 60.2 (±16.2) | 63 (±17.4) | 0.23 | [-0.14, 0.59] | 52.8 (±19.6) | 60.1 (±17.3) | 0.59 | [0.24, 0.95] | -0.12 | [-0.26, 0.04] | 2 | 0.16 |

**Supplementary table 5. Mean, range, and percentage of sub-threshold scores on questionnaires addressing daily difficulties, following ITT principles for the short-term follow-up and PP approach for the long-term follow-up**

|  |  | BASELINE | | | 2 months FOLLOW-UP | | | 8 months FOLLOW-UP | | |
| --- | --- | --- | --- | --- | --- | --- | --- | --- | --- | --- |
|  |  | Mean (*SD*) | Range | % threshold ^2^(*N*) | Mean (*SD*) | Range | % threshold ^2^(*N*) | Mean (*SD*) | Range | % threshold ^2^(*N*) |
| ***Cognitive complaints*** | BRIEF-A (GEC) | 138.2 (±21.1) | 98–186.5 | 39% (48) | 133.3 (±24) | 82–197.5 | 35.3% (43) | 134.3 (±25.8) | 82.5–205 | 38.6% (37) |
|  | BRIEF-A (BRI subscale) | 55.9 (±10.7) | 34.5–78 | 24.4% (30) | 54.3  (±11.5) | 32–81.5 | 18.9% (23) | 54.5 (±11.7) | 30.5–85.5 | 19.8% (19) |
|  | BRIEF-A (MI subscale) | 82.3 (±12.9) | 54.5–114 | 55.3% (68) | 79 (±14.4) | 47–117.5 | 44.3% (54) | 79.7 (±16.1) | 49–119.5 | 51% (49) |
|  | MMQ (composite score) | 94.2 (±18.5) | 41–143.5 | 1.6% (2) | 101.9 (±21.1) | 51.5–165.5 | 0.8% (1) | 102.5 (±23) | 45.5–172.5 | 1% (1) |
|  | MMQ (Satisfaction level) | 20.3 (±11.3) | 3.5–57.5 | 35% (43) | 23.6 (±12.6) | 1.5–61.5 | 25.4% (31) | 24.5 (±14.3) | 0.5–69 | 28.1% (27) |
|  | MMQ (Ability) | 33.7 (±11.6) | 7–63 | 25.2% (31) | 37.1 (±12.7) | 7–67 | 18.9% (23) | 36.1 (±13.7) | 3–63.5 | 18.8% (18) |
|  | MMQ (Strategies) | 40.2 (±11.8) | 16–70.5 | 0% | 41.3 (±11.2) | 13.5–71.5 | 0.8% (1) | 41.9 (±11.1) | 17–66 | 0% |
| ***Fatigue and sleep*** | MFIS (global score) | 62.7 (±13.2) | 13–84 | 95.1% (116) | 56.4 (±17.3) | 3–84 | 85.2% (104) | 53.9 (±18.1) | 9–84 | 80.2% (77) |
|  | PSQI (global score) | 9.2 (±4) | 1–20 | 81.2% (99) | 8.4 (±3.8) | 1–20 | 78.7% (96) | 8.4 (±3.9) | 1–18 | 75% (72) |
| ***Psychological distress and Quality of life*** | OQ-45 (global score) | 70 (±23.1) | 17–119 | 65.6% (80) | 65.9 (±24.4) | 13–127 | 55.7% (68) | 65.2 (±24.9) | 14–126 | 58.3% (56) |
|  | OQ-45 (symptom distress) | 44.7 (±15.8) | 10–80 | 73% (89) | 41.1 (±15.9) | 9–84 | 63.9% (78) | 40.8 (±16.3) | 8–77 | 66.7% (64) |
|  | OQ-45 (interpersonal relations) | 11.9 (±6.4) | 0–31 | 30.3% (37) | 11.9 (±6.6) | 0–34 | 34.4% (42) | 12.4 (±6.8) | 0–33 | 35.4% (34) |
|  | OQ-45 (social role) | 13.4 (±4.8) | 0–31 | 69.7% (85) | 12.9 (±5.3) | 0–26 | 60.7% (74) | 12.1 (±5.1) | 0–24 | 59.4% (57) |
|  | QLSI (global score) | 11.6 (±8.2) | -8.4–37.7 | 80.5% (99) | 8.4 (±7.3) | -14.05–28.54 | 63.9% (78) | 8.6 (±10.7) | -11.6–64.3 | 57.3% (55) |
|  | EQ-5D-5L (VAS score) | 56.4 (±18.3) | 10-90 | / | 61.9 (±17.6) | 11-96 | / | 62.4 (±16.8) | 20-92 | / |
| ***Work and activity*** | Impact on daily activities (% ±*SD*) | 57.8% (±27.4) | 0–100 | / | 53.9% (±29) | 0–100 | / | 38.1% (±28.5) | 0–100 | / |
|  | Impact on work (% ±*SD*) ^1^ | 54.2% (±32.1) | 0–100 | / | 46.6% (±28.6) | 0–99.5 | / | 53.2% (±27.1) | 0–100 | / |

**Notes**. *N*= number of participants (122 at 2-month and 96 at 8-month follow-up).

GEC= Global Executive Composite; BRI= Behavioural Regulation Index; MI= Metacognition Index.

^1^Results for the patients actively employed/studying (*N*=70 at 2-month follow-up and *N*=53 at 8-month follow-up).

^2^Percentage of scores meeting the difficulty threshold.

**Supplementary table 6. Mean, range and percent of sub-threshold scores on questionnaires addressing daily difficulties, following PP principles at short- and long-term follow-up**

|  |  | BASELINE | | | 2 months FOLLOW-UP | | | 8 months FOLLOW-UP | | |
| --- | --- | --- | --- | --- | --- | --- | --- | --- | --- | --- |
|  |  | Mean (*SD*) | Range | % threshold ^2^(*N*) | Mean (*SD*) | Range | % threshold ^2^(*N*) | Mean (*SD*) | Range | % threshold ^2^(*N*) |
| ***Cognitive complaints*** | BRIEF-A (GEC) | 138.1 (±21) | 98–186.5 | 24.4% (30) | 133.5 (±24.1) | 82–197.5 | 38% (41) | 134.3 (±25.8) | 82.5–205 | 38.6% (37) |
|  | BRIEF-A (BRI subscale) | 55.9 (±10.6) | 34.5–78 | 56.1% (69) | 54.3  (±11.6) | 32–81 | 19.4% (21) | 54.5 (±11.7) | 30.5–85.5 | 19.8% (19) |
|  | BRIEF-A (MI subscale) | 82.2 (±12.8) | 54.5–114 | 39.8% (49) | 79.2 (±14.4) | 47–117.5 | 44.4% (48) | 79.7 (±16.1) | 49–119.5 | 51% (49) |
|  | MMQ (composite score) | 94.2 (±18.4) | 41–143.5 | 1.2% (2) | 102.7 (±21.1) | 51.5–165.5 | 0.9% (1) | 102.5 (±23) | 45.5–172.5 | 1% (1) |
|  | MMQ (Satisfaction level) | 20.3 (±11.2) | 3.5–57.5 | 35% (43) | 23.7 (±12.3) | 1.5–61.5 | 25% (27) | 24.5 (±14.3) | 0.5–69 | 28.1% (27) |
|  | MMQ (Ability) | 33.8 (±11.5) | 7–63 | 25.2% (31) | 36.9 (±12.5) | 7–65 | 18.5% (20) | 36.1 (±13.7) | 3–63.5 | 18.8% (18) |
|  | MMQ (Strategies) | 40 (±11.8) | 16–70.5 | 0% | 42 (±10.8) | 21–71.5 | 0% | 41.9 (±11.1) | 17–66 | 0% |
| ***Fatigue and sleep*** | MFIS (global score) | 62.7 (±13.1) | 13–84 | 95% (117) | 55.8 (±18) | 3–84 | 73.8% (90) | 53.9 (±18.1) | 9–84 | 80.2% (77) |
|  | PSQI (global score) | 9.2 (±3.9) | 1–20 | 81.3 % (100) | 8.3 (±3.7) | 1–19 | 69.7% (85) | 8.4 (±3.9) | 1–18 | 75% (72) |
| ***Psychological distress and Quality of life*** | OQ-45 (global score) | 70.1 (±23) | 17–119 | 65.8% (81) | 65.3 (±24) | 13–127 | 46.7% (57) | 65.2 (±24.9) | 14–126 | 58.3% (56) |
|  | OQ-45 (symptom distress) | 44.8 (±15.8) | 10–80 | 73.1% (90) | 40.9 (±15.8) | 9–84 | 57.4% (70) | 40.8 (±16.3) | 8–77 | 66.7% (64) |
|  | OQ-45 (interpersonal relations) | 11.9 (±6.3) | 0–31 | 30% (37) | 11.8 (±6.2) | 0–34 | 27.9% (34) | 12.4 (±6.8) | 0–33 | 35.4% (34) |
|  | OQ-45 (social role) | 13.4 (±4.8) | 0–31 | 69.9% (86) | 12.6 (±5.4) | 0–26 | 55.7% (68) | 12.1 (±5.1) | 0–24 | 59.4% (57) |
|  | QLSI (global score) | 11.6 (±8.2) | –8.3–37.6 | 81.3% (100) | 8.2 (±7.4) | -14.05–28.54 | 55.7% (68) | 8.6 (±10.7) | –11.6–64.3 | 57.3% (55) |
|  | EQ-5D-5L (VAS score) | 56.4 (±18.3) | 10-90 | / | 61.5 (±17.3) | 11-96 | / | 62.4 (±16.8) | 20-92 | / |
| ***Work and activity*** | Impact on daily activities (% ±*SD*) | 57.8% (±27.2) | 0–100 | / | 54.2% (±28.6) | 0–100 | / | 38.1% (±28.5) | 0–100 | / |
|  | Impact on work (% ±*SD*) ^1^ | 54.2% (±32.1) | 0–100 | / | 46.6% (±27.8) | 0–99.5 | / | 53.2% (±27.1) | 0–100 | / |

**Notes**. *N*= number of participants (108 at 2-month and 96 at 8-month follow-up).

GEC= Global Executive Composite; BRI= Behavioural Regulation Index; MI= Metacognition Index.

^1^Results for the patients actively employed/studying (*N*=62 at 2-month follow-up and *N*=53 at 8-month follow-up).

^2^Percentage of scores meeting the difficulty threshold.

**Supplementary table 7. Results at 8-month follow-up for LMM on somatic complaints following a PP approach**

|  | 8-month follow-up | | vs. T0 | | | | vs. 2-month follow-up | | | |
| --- | --- | --- | --- | --- | --- | --- | --- | --- | --- | --- |
|  | AFFECTIVE INTERVENTION | COGNITIVE INTERVENTION | AFFECTIVE INTERVENTION | | COGNITIVE INTERVENTION | | AFFECTIVE INTERVENTION | | COGNITIVE INTERVENTION | |
|  | mean (*SD*) | mean (*SD*) | *d* | CI95 | *d* | CI95 | *d* | IC95 | *d* | CI95 |
| ***QLSI (total score)*** | 9.2 (10.8) | 8 (10.7) | 0.03 | [-0.33, 0.4] | **0.39** | **[-0.03, 0.73]** | 0.38 | [0.02, 0.74] | 0.1 | [-0.25, 0.46] |
| ***MFIS (physical fatigue)*** | 22.6 (9.5) | 24 (8) | **0.64** | **[0.28, 1]** | **0.46** | **[0.11, 0.81]** | 0.1 | [0.27, 0.46] | 0.08 | [-0.27, 0.43] |
| ***MFIS (cognitive fatigue)*** | 26.2 (9.6) | 25.4 (8.6) | **0.62** | **[-0.26, 0.99]** | **0.78** | **[0.42, 1.1]** | -0.09 | [-0.45, 0.28] | 0.2 | [-0.15, 0.55] |
| ***MFIS (psychosocial fatigue)*** | 5 (2.4) | 4.7 (2.4) | 0.02 | [-0.34, 0.38] | **0.6** | **[0.24, 0.95]** | 0.07 | [-0.29, 0.43] | 0.1 | [-0.26, 0.45] |
| ***MFIS (total score)*** | 53.8 (19) | 54.1 (17.4) | **0.66** | **[0.29, 1]** | **0.75** | **[0.39, 1.1]** | 0.01 | [-0.35, 0.37] | 0.16 | [-0.19, 0.52] |
| ***PSQI (total score)*** | 8 (4) | 8.8 (3.9) | 0.05 | [-0.32, 0.41] | 0.35 | [0, 0.7] | -0.18 | [-0.54, 0.18] | -0.03 | [-0.38, 0.32] |
| ***OQ-45 (symptom distress)*** | 39.3 (17.1) | 42.2 (15.5) | 0.06 | [-0.3, 0.4] | **0.6** | **[0.24, 0.96]** | -0.14 | [-0.5, 0.23] | 0.03 | [-0.32, 0.38] |
| ***OQ-45 (interpersonal relation)*** | 11.5 (6.6) | 13.2 (7) | -0.27 | [-0.64, 0.09] | 0.09 | [-0.26, 0.44] | 0.23 | [-0.14, 0.59] | -0.3 | [-0.65, 0.06] |
| ***OQ-45 (social role)*** | 11.8 (5.4) | 12.3 (4.8) | 0.17 | [-0.2, 0.53] | 0.38 | [0.03, 0.73] | 0.15 | [-0.22, 0.51] | 0.14 | [-0.21, 0.49] |
| ***OQ-45 (total score)*** | 62.7 (26.1) | 67.7 (23.6) | 0.01 | [-0.36, 0.37] | 0.52 | [0.17, 0.87] | 0.01 | [-0.35, 0.38] | -0.03 | [-0.38, 0.33] |
| ***WPAI (work impairment)*** | 41.9 (29.5) | 33.5 (27.2) | **0.73** | **[0.28, 1.17]** | **0.68** | **[0.21, 1.15]** | 0.16 | [-0.27, 0.59] | 0.4 | [-0.05, 0.85] |
| ***WPAI (activity impairment)*** | 52.1 (28.9) | 54.3 (25.4) | 0.13 | [-0.23, 0.49] | 0.34 | [-0.02, 0.69] | -0.02 | [-0.38, 0.34] | 0.01 | [-0.34, 0.36] |
| ***EQ-5D-5L (VAS score)*** | 63.9 (19.3) | 61.1 (14) | 0.2 | [-0.17, 0.56] | **0.69** | **[0.34, 1]** | -0.04 | [-0.4, 0.33] | 0.09 | [-0.27, 0.44] |

Note. Significant effects (*p*<0.05) are marked in bold.

**Supplementary table 8. Baseline scores on objective neuropsychological measures per cognitive domain**

| \| Domains \| Total (*n*=122)  Mean (*SD*) \| Cognitive intervention (*n*=63)  Mean (*SD*) \| Affective intervention (*n*=59)  Mean (*SD*) \| \| --- \| --- \| --- \| --- \| \| *Memory (verbal and visuospatial)* \| 0.14 (0.84) \| 0.08 (0.8) \| 0.21 (0.88) \| \| *Attention (Selective attention, divided attention, processing speed and attentional fluctuation)* \| 0.21 (0.59) \| 0.11 (0.54) \| 0.31 (0.63) \| \| *Executive functions (inhibition, flexibility and language)* \| 0.04 (0.78) \| -0.04 (0.79) \| 0.13 (0.76) \| \| *Working memory* \| -0.5 (0.81) \| -0.53 (0.8) \| -0.45 (0.83) \| |  |  |
| --- | --- | --- | --- | --- | --- | --- | --- | --- | --- | --- | --- | --- | --- | --- | --- | --- | --- | --- | --- | --- | --- | --- |
|  |  |  |
|  |  |  |
|  |  |  |
|  |  |  |
|  |  |  |
|  |  |  |
|  |  |  |
|  |  |  |
|  |  |  |
|  |  |  |
|  |  |  |
|  |  |  |
|  |  |  |
|  |  |  |
|  |  |  |
|  |  |  |
|  |  |  |
|  |  |  |
|  |  |  |
|  |  |  |
|  |  |  |
|  |  |  |
|  |  |  |
|  |  |  |
|  |  |  |
|  |  |  |

**Supplementary table 9. Results at 2-month follow-up for LMM on patient’s worst performance per cognitive domain, following a PP approach**

|  | Time effect | | AFFECTIVE INTERVENTION | | | | COGNITIVE INTERVENTION | | | | Time by group interaction | | | |
| --- | --- | --- | --- | --- | --- | --- | --- | --- | --- | --- | --- | --- | --- | --- |
|  | *F* | *p* | Baseline (Mean ± *SD*) | 2 months post-intervention (Mean ± *SD*) | *d* (Time effect) | CI95 | Baseline (Mean ± *SD*) | 2 months post-intervention (Mean ± *SD*) | *d* (Time effect) | CI95 | *d* (interaction effect) | CI95 | *F* | *p* |
| ***Attention*** | 9.9 | 0.002 | -0.5 (±0.67) | -0.36 (±0.72) | 0.24 | [-0.13, 0.6] | -0.7 (±0.58) | -0.39 (±0.75) | 0.57 | [0.21, 0.93] | -0.12 | [-0.3, 0.06] | 1.7 | 0.19 |
| ***Working memory*** | 10.2 | 0.002 | -1.03 (±0.84) | -0.68 (± 1.11) | 0.56 | [0.19, 0.93] | -1 (± 0.82) | -0.79 (± 0.9) | 0.26 | [-0.09, 0.61] | 0.1 | [-0.06, 0.24] | 1.4 | 0.24 |
| ***Executive functions*** | 3.7 | 0.06 | -0.55 (± 0.81) | -0.39 (±0.89) | 0.41 | [0.05, 0.78] | -0.71 (± 0.91) | -0.49 (± 0.95) | 0.08 | [-0.27, 0.44] | 0.12 | [-0.06, 0.3] | 1.7 | 0.20 |
| ***Long-term memory*** | 15.7 | <0.001 | -0.19 (± 1.1) | 0.1 (± 1.04) | 0.33 | [-0.04, 0.7] | -0.48 (± 1.1) | 0.03 (± 0.95) | 0.68 | [0.32, 1.04] | -0.12 | [-0.28, 0.06] | 1.9 | 0.18 |

**Supplementary table 10. Results at 8-month follow-up for LMM on patient’s worst performance per cognitive domain following a PP approach**

|  | 8-month follow-up | | vs. T0 | | | | vs. 2-month follow-up | | | |
| --- | --- | --- | --- | --- | --- | --- | --- | --- | --- | --- |
|  | AFFECTIVE INTERVENTION | COGNITIVE INTERVENTION | AFFECTIVE INTERVENTION | | COGNITIVE INTERVENTION | | AFFECTIVE INTERVENTION | | COGNITIVE INTERVENTION | |
|  | mean (± *SD*) | mean (± *SD*) | *d* | CI95 | *d* | CI95 | *d* | CI95 | *d* | CI95 |
| ***Attention*** | -0.22 (±0.8) | -0.15 (± 0.8) | 0.43 | [0.07,0.79] | **0.92** | **[0.56,1.3]** | 0.18 | [-0.18, 0.55] | 0.32 | [-0.04, 0.67] |
| ***Working memory*** | -0.74 (± 1.2) | -0.58 (± 0.9) | 0.41 | [0.05, 0.77] | **0.54** | **[0.19, 0.9]** | -0.19 | [-0.55, 0.17] | 0.27 | [-0.09, 0.62] |
| ***Executive functions*** | -0.38 (± 1) | -0.36 (± 0.9) | 0.08 | [-0.28, 0.44] | 0.33 | [-0.03, 0.68] | -0.02 | [-0.38, 0.34] | 0.31 | [-0.05, 0.66] |
| ***Long-term memory*** | 0 (± 1.1) | 0.13 (± 1) | 0.21 | [-0.15, 0.58] | **0.67** | **[0.31, 1]** | -0.14 | [-0.5, 0.23] | -0.04 | [-0.39, 0.31] |

Note. Significant effects (*p*<0.05) are marked in bold.

**Supplementary table 11. Results of linear regression analysis**

Linear regressions analyses were conducted on BRIEF-A and MMQ scores (composite scores and subscale scores), with time between first SARS-CoV-2 infection and study inclusion (T0), and time between first infection and short-term follow-up evaluation (T1).

|  | Time between first infection and baseline evaluation | | Time between first infection and short-term evaluation | |
| --- | --- | --- | --- | --- |
|  | *t* | *p* | *t* | *p* |
| ***BRIEF-A (BRI)*** | -0.5 | 0.62 | -0.7 | 0.49 |
| ***BRIEF-A (MI)*** | 0.9 | 0.37 | -0.1 | 0.91 |
| ***BRIEF-A (GEC)*** | 0.3 | 0.77 | -0.4 | 0.69 |
| ***MMQ (Satisfaction)*** | 0.2 | 0.81 | -0.7 | 0.47 |
| ***MMQ (Ability)*** | 0.7 | 0.47 | 0.5 | 0.62 |
| ***MMQ (Strategies)*** | -1.7 | 0.09 | -0.8 | 0.45 |
| ***MMQ (composite score)*** | -0.4 | 0.65 | -0.5 | 0.59 |

Notes. GEC= Global Executive Composite; BRI= Behavioural Regulation Index; MI= Metacognition Index.

**Supplementary table 12. Summary of statistically significant results for time effect from LMMs following a per-protocol approach**

|  |  | T1 vs. T0 | | T2 vs. T0 | | T2 vs. T1 | |
| --- | --- | --- | --- | --- | --- | --- | --- |
|  |  | *Affective intervention* | *Cognitive intervention* | *Affective intervention* | *Cognitive intervention* | *Affective intervention* | *Cognitive intervention* |
| *Cognitive performance* | *Attention* | / | Improvement (d=0.57) | / | Improvement (d=0.92) | / | / |
|  | *Working memory* | Improvement (d=0.56) | / | / | Improvement (d=0.54) | / | / |
|  | *Executive functions* | / | / | / | / | / | / |
|  | *Long-term memory* | / | Improvement (d=0.68) | / | Improvement (d=0.67) | / | / |
| *Cognitive complaints* | ***BRIEF-A (GEC)*** | **Improvement (d=0.51)** | **Improvement (d=0.54)** | / | Improvement (d=0.55) | / | / |
|  | ***MMQ (composite score)*** | **Improvement (d=0.54)** | **Improvement (d=0.49)** | Improvement (d=0.54) | Improvement (d=0.85) | / | / |
|  | *BRIEF-A (BRI)* | Improvement (d=0.45) | Improvement (d=0.41) | / | Improvement (d=0.62) | / | / |
|  | *BRIEF-A (MI)* | Improvement (d=0.59) | Improvement (d=0.43) | / | Improvement (d=0.59) | / | / |
|  | *MMQ (Satisfaction)* | Improvement (d=0.44) | Improvement (d=0.53) | / | Improvement (d=0.45) | / | / |
|  | *MMQ (Ability)* | Improvement (d=0.51) | Improvement (d=0.64) | Improvement (d=0.56) | Improvement (d=0.88) | / | / |
|  | *MMQ (Strategies)* | / | / | / | / | / | / |
| *Fatigue* | *MFIS (physical fatigue)* | Improvement (d=0.55) | Improvement (d=0.38) | Improvement (d=0.64) | Improvement (d=0.46) | / | / |
|  | *MFIS (cognitive fatigue)* | Improvement (d=0.64) | Improvement (d=0.52) | Improvement (d=0.62) | Improvement (d=0.78) | / | / |
|  | *MFIS (psychosocial fatigue)* | / | Improvement (d=0.48) | / | Improvement (d=0.6) | / | / |
|  | *MFIS (total score)* | Improvement (d=0.6) | Improvement (d=0.54) | Improvement (d=0.66) | Improvement (d=0.75) | / | / |
| *Sleep problems* | *PSQI (total score)* | / | Improvement (d=0.38) | / | / | / | / |
| *Quality of life* | *QLSI (total score)* | Improvement (d=0.39) | Improvement (d=0.54) | / | / | / | / |
|  | *EQ-5D-5L (VAS score)* | / | Improvement (d=0.59) | / | Improvement (d=0.69) | / | / |
| *Psychological distress* | *OQ-45 (symptom distress)* | / | Improvement (d=0.51) | / | Improvement (d=0.6) | / | / |
|  | *OQ-45 (interpersonal relations)* | Improvement (d=0.45) | / | / | / | / | / |
|  | *OQ-45 (social role)* | / | / | / | / | / | / |
|  | *OQ-45 (total score)* | / | / | / | / | / | / |
| *Work and activity impairment* | *Work impairment* | Improvement (d=0.57) | / | Improvement (d=0.73) | Improvement (d=0.68) | / | / |
|  | *Activity impairment* | / | / | / | / | / | / |

Notes. GEC= Global Executive Composite; BRI= Behavioural Regulation Index; MI= Metacognition Index. d= Cohen’s d. No significant effect reflected a deterioration. Primary outcomes are marked in bold.
